# Medicare Advantage's Provision of Expanded Supplemental Benefits and Acute Care Utilization

**DOI:** 10.1111/1475-6773.70136

**Published:** 2026-05-29

**Authors:** Jeah Jung, Ge Song, Roger Feldman, Caroline Carlin, Daniel Polsky, Hyunjee Kim

**Affiliations:** ^1^ Department of Health Administration and Policy, College of Public Health George Mason University Fairfax Virginia USA; ^2^ Division of Health Policy and Management, School of Public Health University of Minnesota Minneapolis Minnesota USA; ^3^ Department of Family Medicine and Community Health University of Minnesota Medical School Minneapolis Minnesota USA; ^4^ Department of Health Policy and Management Johns Hopkins Bloomberg School of Public Health Baltimore Maryland USA; ^5^ Center for Health Systems Effectiveness, School of Medicine Oregon Health and Science University Portland Oregon USA

## Abstract

**Objective:**

To examine whether Medicare Advantage (MA) plans' provision of expanded supplemental benefits reduces enrollees' acute care utilization. Expanded supplemental benefits included non‐medical primarily health‐related (PHR) benefits such as in‐home support services, starting in 2019, and Special Supplemental Benefits for the Chronically Ill (SSBCI) such as food security and housing quality benefits, starting in 2020.

**Study Setting and Design:**

Quasi‐experimental design using staggered difference‐in‐differences models. We created indicators for offering the following benefits: Any expanded PHR, ≥ 2 expanded PHR, any SSBCI, and ≥ 2 SSBCI. Acute care utilization was measured by three binary indicators of adverse health events: annual emergency department (ED) use, hospitalizations, and re‐admissions.

**Data Sources and Analytic Sample:**

We used 2017–2022 MA encounter data from a random 20% sample of enrollees. We estimated separate models for each benefit indicator, for all non‐dual and all dual‐eligible enrollees, and for highly frail patients within each non‐dual and dual‐eligible group. We used propensity score matching to balance baseline characteristics between treatment and control groups.

**Principal Findings:**

Offering expanded supplemental benefits did not generally reduce acute care utilization in all non‐dual or all dual‐eligible enrollees. A few significant effects were relatively small or were not robust to potential differential trends between benefit‐offering and not‐offering plans. However, among highly frail non‐dual enrollees, offering any PHR reduced re‐admissions by −2.82 percentage points (95% CI: −4.78, −0.86), offering ≥ 2 expanded PHR reduced hospitalizations by −1.79 percentage points (95% CI: −2.61, −0.98) and ED use by −1.76 percentage points (95% CI: −2.38, −1.14), and offering ≥ 2 SSBCI reduced ED use by −3.51 percentage points (95% CI: −4.96, −2.05) and hospitalizations by −2.05 percentage points (95% CI: −3.12, −0.099).

**Conclusions:**

Provision of expanded supplemental benefits may reduce acute care utilization for certain enrollees. As spending on MA supplemental benefits rises, continued efforts are needed to assess impacts of those benefits.

## Callout Box

**What Is Known on This Topic:**

- Medicare Advantage (MA) supplemental benefits were expanded to include non‐medical primarily health‐related (PHR) benefits in 2019 and Special Supplemental Benefits for the Chronically Ill (SSBCI) in 2020.
- Expanded supplemental benefits are expected to maintain or improve enrollees' health and thereby reduce acute care utilization, such as emergency department use, hospitalizations, and re‐admissions.
- MA plans' adoption of expanded supplemental benefits has steadily increased, but little is known about whether it reduces enrollees' acute care utilization.

**What This Study Found:**

- This is the first study to examine the impacts of MA plans offering expanded supplemental benefits on enrollees' acute care utilization.
- Offering expanded supplemental benefits did not generally reduce emergency department use, hospitalizations, or re‐admissions in either all non‐dual or all dual‐eligible enrollees, although we did not observe actual benefit use.
- For highly frail non‐dual enrollees, offering any PHR reduced re‐admissions, offering ≥ 2 expanded PHR reduced ED use and hospitalizations, and offering ≥ 2 SSBCI reduced ED use and hospitalizations.

## 1 Introduction

Medicare Advantage (MA) plans provide supplemental benefits that are not covered in traditional Medicare. These benefits are largely financed through rebates generated when capitated payments to MA plans exceed plans' cost for basic Medicare benefits. MA plans may use rebates to offer supplemental benefits, reduce premiums, or reduce cost sharing. Rebates have significantly increased in recent years, reaching $86 billion in 2025, up from $21 billion in 2018 [1]. MA plans have increasingly used rebates to provide supplemental benefits, allocating 40% of rebates to supplemental benefits in 2025, compared with 20% in 2018 [1].

This growth in spending on supplemental benefits coincided with a policy change that granted MA plans flexibility in designing supplemental benefits. Supplemental benefits were traditionally limited to primarily health‐related (PHR) benefits, such as dental or vision services, which incur direct medical costs. In 2019, the definition of PHR was re‐interpreted to include non‐medical supportive care, such as in‐home support services, adult daycare, and home palliative care [2]. Starting in 2020, MA plans could offer non‐PHR social service benefits, known as Special Supplemental Benefits for the Chronically Ill (SSBCI), to address health‐related social needs in enrollees with chronic conditions. SSBCIs include food/produce, meals beyond post‐discharges, non‐medical transportation, pest control, indoor air quality, structural home modification, living supports (e.g., utility coverage), and social needs benefits [3]. We refer to expanded PHR benefits and SSBCI together as expanded supplemental benefits.

This expansion of MA supplemental benefits was the first initiative to incorporate non‐medical benefits into Medicare. In part, it was a response to the growing recognition that supportive care and social services play an important role in maintaining or improving individuals' health [4]. Medicare attempted to incorporate this recognition by leveraging MA plans' supplemental benefits [5]. Policy makers expected that expanded supplemental benefits would reduce adverse acute health events such as emergency department (ED) visits, which can be caused by poor health [3, 6]. Meeting patients' needs for health‐related social services could affect individuals' health and thereby reduce acute care use through direct and indirect pathways. Directly, appropriate meals foster physical strength and prevent disease progression [7, 8], adequate housing quality (e.g., indoor air quality) helps prevent worsening of respiratory conditions [9, 10], and home modification reduces the risk of falls [11, 12, 13]. Indirectly, providing social services frees up patients' resources for health care, such as medications and care management services [8], and it increases material and mental stability, which lowers stress and empowers patients to seek appropriate care [14]. Use of appropriate management services can prevent disease exacerbations.

MA plans supported this expansion of supplemental benefits, discussing the value of meeting social needs, such as keeping enrollees in the community as much as possible [6, 15]. Plans also indicated that the ability to increase or retain members would drive them to invest in expanded supplemental benefits [15]. In fact, plans in more competitive markets were more likely to offer SSBCI to attract certain enrollee groups [16].

MA plans have increasingly adopted expanded supplemental benefits. Our analysis of MA Plan Benefits data [17] indicated that about 18% of plans provided at least one expanded PHR by 2025 and 36% provided at least one SSBCI. Certain benefits, such as food/produce benefits, grew faster than others [18]. Despite this growth, few studies have evaluated the impacts of MA plans' provision of expanded supplemental benefits for beneficiaries. MA plans offering any expanded supplemental benefit had higher enrollee plan ratings than plans that did not [19], but similar disenrollment rates [20]. Little is known about whether offering expanded supplemental benefits reduces adverse acute care utilization, such as ED use or hospitalizations, which is an intended outcome of those benefits. This evidence gap constrains efforts to evaluate and refine policies regarding MA supplemental benefits [1]. To address this gap, we examined the impacts of MA plans' provision of expanded supplemental benefits on acute care utilization.

## 2 Methods

### 2.1 Data and Study Population

The primary data source was 2017–2022 national MA encounter data for inpatient and outpatient services. We used MA Plan Benefit Files to obtain information about supplemental benefits provided by each plan—i.e., each benefit package within a contract. Medicare Master Beneficiary Summary Files provided information on each enrollee's demographics, ZIP code of residence, Medicare‐Medicaid dual eligibility status, and MA coverage. We used the American Community Survey for ZIP‐level socioeconomic factors [21], Rural–Urban Commuting Area Codes for rurality of the area [22], and the Area Health Resource File for county‐level health care resources [23].

The study population comprised a random 20% sample of MA enrollees. We required beneficiaries to have continuous enrollment in MA in a given year and to reside in the 50 states or D.C. We excluded long‐term (> 100 days) nursing home stayers, enrollees with Medicare eligibility due to end‐stage renal disease, and enrollees in special types of plans, such as employer‐sponsored, cost, PACE, or Chronic‐condition Special Needs Plans (C‐SNPs). We included enrollees only from MA contracts with highly complete encounter records, to account for incompleteness of encounter data [24]. Appendix Table A1 reports the steps to identify the study sample and the associated numbers.

### 2.2 Expanded Supplemental Benefit Indicators and Analysis Samples

We created two indicators that represent provision of expanded PHR benefits: (1) offering any expanded PHR; and (2) offering ≥ 2 expanded PHR benefits. The latter captures a greater extent of benefit provision. By 2022, 23.4% of plans offered any expanded PHR, and 7.8% offered two or more (Appendix Figure A1.1). More detailed categorizations (e.g., three or four benefits) were not feasible due to limited sample sizes. The samples for these analyses were the study population described above.

Next, we created two indicators related to SSBCI: (3) offering any SSBCI; and (4) offering ≥ 2 SSBCIs. By 2022, 22.1% of plans offered any SSBCI, and 11.3% offered two or more (Appendix Figure A1.1). The samples for these analyses comprised enrollees with a chronic condition(s) because only the chronically ill are eligible for SSBCIs. We used chronic conditions from twelve condition categories (Appendix A1), which meet the chronic condition criterion for SSBCI eligibility identified by the Centers for Medicare and Medicaid Services (CMS) [25, 26].

Our primary analyses used indicators for offering expanded PHR or SSBCI benefits because the rates of adoption for specific benefits were relatively low during the study period (the first 4 years of benefit offering). However, about 75% of plans with expanded PHR benefits in 2022 offered in‐home support services, and over 80% of plans offering SSBCI included a food security benefit (food/produce or meals beyond post‐discharges). We performed a secondary analysis, creating indicators for these specific benefits that were more commonly adopted and using enrollees with conditions relevant to the benefit in question as the analysis sample (see the sensitivity analyses section).

### 2.3 Outcomes

Outcomes were binary (yes/no) indicators of annual adverse acute care utilization: any ED visit, hospitalization, and 30‐day re‐admission [27].

### 2.4 Covariates

Covariates included plan‐county fixed effects to control for time‐invariant plan‐county specific attributes, and year fixed effects to control for common secular trends in outcomes across all MA plans. We also controlled for enrollee, plan, and area factors. Enrollee characteristics included age, sex, race/ethnicity (Asian, Hispanic, Non‐Hispanic Black, Non‐Hispanic White, and Others; using the Research Triangle Institute's race classification) [28], a claims‐based frailty score [29], and 22 chronic condition indicators constructed from the Chronic Condition Warehouse (CCW) algorithm [30]. We controlled for plan factors with indicators for time‐varying supplemental benefits (e.g., medical transportation) other than the benefit in question. Area‐level covariates included the median household income^,^ percent college educated, rurality, and the numbers of hospital beds and doctors.

### 2.5 Model

We used a quasi‐experimental design to leverage MA plans' staggered adoption of expanded supplemental benefits. Specifically, we estimated a staggered difference‐in‐differences (DiD) model to isolate the effect of benefit provision from time‐invariant confounders and common secular trends. Staggered DiD accommodates heterogeneity in the treatment effect across cohorts with different timing of benefit adoption [31]. The model is written as follows:

(1) $$Y_{\text{ijkt}}=\alpha_{0}+\sum_{c}\sum_{l=-L^{\mathrm{pre}}}^{L^{\text{post}}}\delta_{c,l}C_{\mathrm{ijk}}^{c}{\text{Benefit}}_{\mathrm{jt}}^{l}+\lambda_{\mathrm{jk}}+{\text{Year}}_{t}+\gamma \cdot X_{\text{ijkt}}+\mu \cdot Z_{\mathrm{jt}}+\epsilon_{\text{ijkt}}$$

Subscripts i, j, k, and t represent enrollee, plan, county, and year. c denotes the cohort defined by plan $j^{\prime }$s benefit adoption time for enrollee i in county k, and l is a count of years from benefit offering. The reference period is the last pre‐benefit offering year (omitted if l=−1). $L^{\mathrm{pre}}$ and $L^{\text{post}}$ are the maximum counts of pre‐ and post‐offering years. $Y_{\text{ijkt}}$ is an outcome, $C_{\mathrm{ijk}}^{c}$ = 1 if enrollee i in plan j and county k belongs to cohort c and 0 otherwise and ${\text{Benefit}}_{\mathrm{jt}}^{l}$ equals one for a benefit‐offering plan during year t when t corresponds to time distance l, and zero for all others. $\lambda_{\mathrm{jk}}$ are plan‐county fixed effects, ${\text{Year}}_{t}$ are year fixed effects, $X_{\text{ijkt}}$ is a vector of patient and area factors, $Z_{\mathrm{jt}}$ is a vector of time‐varying plan attributes, such as indicators of other supplemental benefits, and $\epsilon_{\text{ijkt}}$ is an error term.

$\delta_{c,l}$ is the average effect for cohort c at relative time l. The aggregate effect across all cohorts at time l is a weighted average of the cohort‐specific effects: $\theta_{l}=\sum_{c}w_{c}\delta_{c,l}$ where $w_{c}$ is the share of treated observations in cohort c. We obtained the overall post‐offering effect as a weighted average of the post‐period coefficients, $\theta_{l}$, where the weight is the proportion of treated enrollee‐time observations at time l, using a linear combination command in Stata.

### 2.6 Treatment (Benefit‐Offering) and Control (Not‐Offering) Plans

Treatment plans adopted an expanded supplemental benefit(s) during the study period. Treatment plans must have at least one pre‐benefit offering period. We included treatment plans only up to benefit‐offering years—i.e., if a plan dropped the benefit in year *t*, we excluded the plan from year *t* onward. Control plans never adopted an expanded supplemental benefit during the study period.

### 2.7 Analytic Challenges

Analyzing the impact of MA plans' benefit provision is challenging because of sample selection: plans with certain covered populations (e.g., plans with a high share of non‐White enrollees) may selectively decide to offer expanded benefits [16]; and county‐specific factors (e.g., the breadth of provider networks) within a plan may also be associated with benefit adoption and outcomes. Plan‐county fixed effects in the DiD model control for time‐invariant plan‐county characteristics associated with this selection. However, they do not control for patients' non‐random plan choices: patients with certain unobserved factors that are correlated with outcomes may selectively enroll in benefit‐offering plans. We addressed this issue in three ways. First, we limited the analysis to enrollees who stayed in the same plan during the pre‐ and post‐offering periods, provided they are in the sample. These “stayers” chose their plan prior to the plan's benefit offering and received the “treatment” (benefit offering) when their plan adopted the benefit after the exogenous policy change. In other words, *plan choice was pre‐determined before benefit offering*. Second, we required enrollees to contribute only to either the treatment or control group—i.e., enrollees in the control group were never exposed to a benefit‐offering plan during the study period. Third, we ensured similar characteristics between treatment and control groups by selecting control enrollees based on propensity score matching at the baseline period—1 year prior to benefit offering—separately for each analysis. Appendix A2 describes the requirement for plan enrollment and the patient matching process in detail.

### 2.8 Analysis

We used linear probability estimation because it readily includes fixed effects and has straightforward interpretations of DiD effects. We clustered standard errors within plan‐counties. We analyzed dual‐eligible and non‐dual enrollees separately because patterns of service use and plan enrollment may differ between the two groups.

Additionally, we estimated the model only with enrollees in the top tercile (33%) of the frailty score at the baseline period, within each group by dual‐eligibility status. These analyses focus on individuals who have potentially more exposure to the benefit. Highly frail enrollees have a high likelihood of receiving expanded supplemental benefits because those benefits target high‐risk enrollees [20, 32]. For example, patients' need for intensive care coordination is a requirement for SSBCI eligibility [3]. Thus, the analyses of highly frail enrollees limit the attenuation of benefit impacts from our approach that estimates the effects of plans' *benefit offering* on acute care use *among potential recipients*.

### 2.9 Checking Parallel Trends

We tested the parallel trends assumption by estimating average differential effects in outcomes during the pre‐treatment period from Equation (1), using the same approach for estimating overall post‐offering DiD effects. When we identified differential pre‐treatment trends between treatment and control groups while showing significant staggered DiD effects during the post‐treatment period, we examined the robustness of DiD estimates to differential trends using *HonestDiD* [33]. This method allows post‐treatment deviations bounded, in magnitude, to the deviations observed in the pre‐treatment period and it constructs confidence intervals (CIs) that are valid under those bounded deviations. If CIs of *HonestDiD* estimates include zero when a small deviation is allowed, that would imply DiD estimates are not robust to potential violation of the parallel trends assumption.

### 2.10 Secondary Analyses

We estimated the model using indicators for offering a single benefit or benefit group that showed steady growth during the study period (Appendix Figure A1.2): (1) in‐home support services; (2) food security benefits (food/produce or meals not limited to post‐discharges); and (3) housing quality benefits (pest control, indoor air quality, or structural home modification). We limited the sample for the analysis of food security benefits to enrollees with a food‐sensitive condition—diabetes, cardiovascular disease, or chronic heart failure. The samples for the other two analyses comprised enrollees with chronic conditions.

## 3 Results

Table 1 presents enrollee characteristics of the study sample. Before matching, the sample included 1,878,404 non‐dual‐ and 483,711 dual‐eligible enrollee‐years in benefit‐offering plans and 7,945,673 non‐dual‐ and 1,131,480 dual‐eligible enrollee‐years in not‐offering plans. Enrollees in benefit‐offering plans were more likely to be Hispanic in both non‐dual and dual‐eligible groups, compared with enrollees in not‐offering plans. Among dual‐eligible individuals, enrollment in coordination‐only Dual‐Eligible Special Needs Plans (D‐SNPs) was higher in benefit‐offering plans than in not‐offering plans, while integrated D‐SNP enrollment was relatively similar. After the matching, the sample consisted of 1,837,571 non‐dual and 455,186 dual enrollee‐years in benefit‐offering plans and 1,832,329 non‐dual and 363,152 dual enrollee‐years in not‐offering plans. Enrollee characteristics between benefit‐offering and not‐offering plans were similar in the matched sample. Eight percent of non‐dual‐ and 23% of dual‐eligible enrollees were Hispanics in both benefit‐offering and not‐offering groups. About half of dual‐eligible enrollees were in coordination‐only D‐SNPs, and about 16% were in integrated D‐SNPs, with little difference between benefit‐offering and not‐offering groups. Appendix Tables A2.1 through A5.2 report descriptive statistics and standardized differences for all study variables by analysis sample.

**TABLE 1:** Enrollee characteristics of the study population.

	Non‐dual eligible enrollees, Mean (SD) or %				Dual eligible enrollees, Mean (SD) or %			
	Before matching		After matching		Before matching		After matching	
	Benefit offering plans	Not‐offering plans	Benefit offering plans	Not‐offering plans	Benefit offering plans	Not‐offering plans	Benefit offering plans	Not‐offering plans
Female	55.8%	54.7%	55.7%	55.7%	63.4%	61.1%	63.3%	63.6%
Age								
Age < 65	6.5%	5.3%	6.4%	5.9%	32.6%	30.8%	32.8%	32.8%
65 ≤ Age < 70	23.4%	25.6%	23.4%	21.3%	18.9%	22.4%	18.8%	18.3%
70 ≤ Age < 75	27.0%	25.8%	27.1%	28.2%	18.4%	16.3%	18.4%	18.3%
75 ≤ Age < 80	20.0%	19.1%	20.0%	21.2%	13.2%	11.9%	13.2%	13.4%
80 ≤ Age < 85	12.5%	12.2%	12.5%	12.9%	8.9%	8.7%	8.9%	9.0%
Age ≥ 85	10.8%	12.1%	10.7%	10.5%	8.1%	9.9%	8.0%	8.2%
Race								
Asian	3.7%	3.3%	3.7%	3.3%	6.3%	8.4%	6.4%	7.2%
Hispanic	8.5%	6.4%	8.4%	7.9%	23.9%	21.5%	23.6%	23.2%
Non‐Hispanic Black	6.7%	9.6%	6.7%	7.3%	20.5%	22.5%	20.7%	21.2%
Non‐Hispanic White	78.4%	77.8%	78.5%	78.6%	47.3%	45.0%	47.3%	46.3%
Other race^a^	2.8%	2.9%	2.8%	2.9%	2.0%	2.7%	2.0%	2.2%
Frailty score, Mean (SD)	0.15 (0.06)	0.16 (0.06)	0.15 (0.06)	0.15 (0.06)	0.18 (0.07)	0.19 (0.08)	0.18 (0.07)	0.18 (0.07)
Chronic conditions^b^								
Anemia	13.9%	16.8%	13.9%	14.7%	21.0%	24.1%	21.1%	22.1%
Arthritis^c^	29.6%	31.8%	29.6%	30.7%	39.4%	36.7%	39.4%	38.7%
Cancer								
Breast	3.8%	4.2%	3.8%	4.0%	3.2%	3.3%	3.2%	3.4%
Colon	1.3%	1.5%	1.3%	1.3%	1.3%	1.6%	1.3%	1.4%
Endometrial	0.5%	0.6%	0.5%	0.5%	0.5%	0.6%	0.5%	0.6%
Lung	0.8%	1.2%	0.8%	0.8%	1.0%	1.5%	1.0%	1.0%
Prostate	4.1%	4.5%	4.1%	4.2%	2.2%	2.3%	2.2%	2.1%
Urologic	0.6%	0.8%	0.6%	0.6%	0.6%	0.6%	0.6%	0.6%
Cardiovascular disease								
AMI	0.7%	1.1%	0.7%	0.9%	1.0%	1.7%	1.0%	1.3%
Atrial fibrillation	10.6%	12.6%	10.6%	10.7%	8.4%	10.5%	8.4%	8.6%
Ischemic heart disease	16.8%	19.1%	16.7%	17.2%	18.3%	19.7%	18.4%	18.3%
Chronic kidney disease	19.8%	19.1%	19.7%	18.8%	24.2%	22.8%	24.3%	22.5%
Dementia								
Alzheimer's	1.2%	1.9%	1.2%	1.2%	1.8%	2.8%	1.7%	1.8%
Non‐Alzheimer's	3.4%	5.4%	3.4%	3.6%	5.6%	8.5%	5.5%	5.8%
Depress	15.6%	15.3%	15.5%	15.1%	31.9%	28.6%	32.0%	30.2%
Diabetes	25.5%	27.2%	25.5%	25.9%	38.1%	38.8%	38.2%	38.6%
Heart failure	8.9%	10.5%	8.9%	8.7%	13.4%	15.4%	13.4%	13.5%
Lung diseases								
Asthma	5.9%	6.8%	5.8%	6.0%	11.5%	12.2%	11.7%	12.3%
COPD	12.6%	12.5%	12.5%	12.0%	23.8%	20.8%	23.9%	21.8%
Pneumonia	2.5%	3.9%	2.5%	2.9%	4.4%	6.6%	4.3%	5.1%
Parkinson's	1.0%	1.4%	1.0%	1.0%	1.2%	1.4%	1.1%	1.1%
Stroke	3.2%	3.9%	3.2%	3.4%	4.0%	5.4%	4.0%	4.6%
Enrollment in D‐SNPs								
Co‐only D‐SNPs	NA	NA	NA	NA	51.3%	38.7%	52.7%	49.7%
Integrated D‐SNPs^d^	NA	NA	NA	NA	17.0%	15.6%	16.7%	15.8%
No. of enrollees	489,534	2,312,069	470,146	470,146	137,992	445,932	122,318	122,318
No. of enrollee‐year	1,878,404	7,945,673	1,837,571	1,832,329	483,711	1,131,480	455,186	363,152

Abbreviations: AMI, acute myocardial infarction; COPD, chronic obstructive pulmonary disease; D‐SNPs, dual‐eligible special needs plans; SD, standard deviation.

^a^ Other race is a category defined in the Master Beneficiary Summary File (MBSF).

^b^ Following the Chronic Condition Warehouse algorithm, 2 years of data are used to construct all conditions except for AMI, pneumonia, and stroke.

^c^ Arthritis includes rheumatoid arthritis and osteoarthritis.

^d^ Integrated D‐SNPs include fully integrated dual‐eligible special needs plans (FIDE) or highly integrated dual‐eligible special needs plans (HIDE).

**FIGURE 1:**
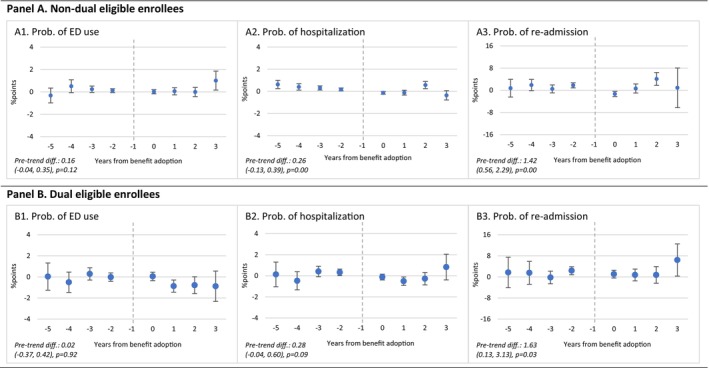
Event‐study plots from the analysis of offering any expanded PHR benefit. Panel A. Non‐dual eligible enrollees. Panel B. Dual eligible enrollees. %points, percentage points; ED, emergency department; PHR, primarily health‐related; pre‐trend diff., pre‐treatment trends difference. Pre‐trend diff. reports the average differential change in the outcome between benefit‐offering plans and not‐offering plans during the pre‐treatment period. The outcome was the annual probability of having an adverse health event (ED visit, hospitalization, or re‐admission). The unit of *Y*‐axis is one percentage point. 95% confidence intervals are in parentheses, and the *p*‐value corresponds to the parallel trends test during the pre‐treatment period. In graphs, error bars indicate the 95% CIs. Year −1 is the omitted reference period.

**FIGURE 2:**
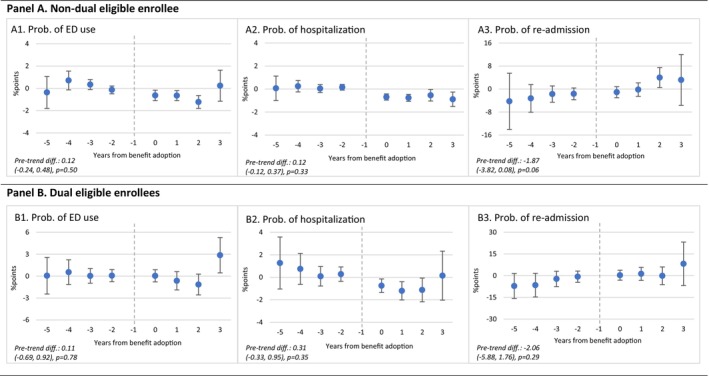
Event‐study plots from the analysis of offering ≥ 2 expanded PHR benefits. Panel A. Non‐dual eligible enrollee. Panel B. Dual eligible enrollees. %points, percentage points; ED, emergency department; PHR, primarily health‐related; pre‐trend diff., pre‐treatment trends difference. Pre‐trend diff. reports the average differential change in the outcome between benefit‐offering plans and not‐offering plans during the pre‐treatment period. The outcome was the annual probability of having an adverse health event (ED visit, hospitalization, or re‐admission). The unit of *Y*‐axis is one percentage point. 95% confidence intervals are in parentheses, and the *p*‐value corresponds to the parallel trends test during the pre‐treatment period. In graphs, error bars indicate the 95% CIs. Year −1 is the omitted reference period.

Table 2 reports the estimates of the overall treatment effects from staggered DiD models. Along with overall DiD effects, we plot event‐study estimates in Figure 1 for offering any expanded PHR benefit and in Figure 2 for offering ≥ 2 expanded PHR benefits. We report event‐study plots for offering any SSBCI in Appendix Figure A2 and for offering ≥ 2 SSBCI in Appendix Figure A3, due to space constraints.

**TABLE 2:** Difference‐in‐differences estimates for MA's provision of expanded supplemental benefits.

	Non‐dual eligible enrollees			Dual eligible enrollees		
	*N*	Baseline mean (%)	Estimate (95% CI), % point	*N*	Baseline mean (%)	Estimate (95% CI), % point
Offering any expanded PHR benefit						
Prob. of ED use^a^	3,664,854	22.0	0.02 (−0.16, 0.21)	815,275	39.4	−0.08 (−0.47, 0.31)
Prob. of hospitalization^a^	3,664,854	9.5	−0.11 (−0.23, 0.00)	815,275	15.8	−0.15 (−0.42, 0.12)
Prob. of re‐admission^a^	130,086	12.4	−1.12 (−2.02, −0.22)*	52,738	17.1	1.06 (−0.35, 2.47)
Offering ≥ 2 expanded PHR benefits						
Prob. of ED use	1,018,693	22.9	**−0.67 (−1.11, −0.23)** **	212,209	38.9	−0.13 (−0.98, 0.72)
Prob. of hospitalization	1,018,693	10.1	**−0.69 (−0.96, −0.43)** ***	212,209	15.9	**−0.84 (−1.44, −0.23)** **
Prob. of re‐admission	32,551	13.4	−0.62 (−2.47, 1.23)	12,061	18.5	0.48 (−2.90, 3.87)
Offering any SSBCI						
Prob. of ED use	799,801	27.8	0.07 (−0.39, 0.53)	487,803	42.9	0.09 (−0.46, 0.64)
Prob. of hospitalization	799,801	13.6	−0.31 (−0.66, 0.03)	487,803	18.6	0.18 (−0.22, 0.58)
Prob. of re‐admission	39,071	14.2	0.68 (−1.41, 2.78)	36,812	18.0	3.26 (1.21, 5.31)**
Offering ≥ 2 SSBCI						
Prob. of ED use	432,447	28.9	−0.23 (−0.93, 0.46)	264,278	43.3	0.03 (−0.67, 0.73)
Prob. of hospitalization	432,447	14.1	−0.25 (−0.73, 0.23)	264,278	18.8	0.31 (−0.22, 0.85)
Prob. of re‐admission	20,847	13.5	−1.87 (−4.87, 1.13)	19,512	18.3	1.30 (−1.26, 3.85)

*Note:* Estimates of the overall treatment effects obtained from staggered difference‐in‐differences models using the matched sample, controlling for plan‐county fixed effects, year dummies, enrollee demographics and health risks, area socio‐economic factors and health resource variables, and time‐varying plan attributes, including supplemental benefits other than the benefit in question. Standard errors are clustered at the plan level. Bolded results are statistically significant and supported by parallel trends during the pre‐treatment period.

Abbreviations: %points, percentage points; CI, confidence interval; ED, emergency department; MA, Medicare Advantage; PHR, primarily health‐related; SSBCI, special supplemental benefits for the chronically Ill.

^a^ Annual probability of having an ED visit, hospitalization, or re‐admission.

***p‐value < 0.001, **p‐value < 0.01, and *p‐value < 0.05.

The top panel of Table 2 indicates that MA plans' offering any expanded PHR did not reduce the annual probability of an ED visit or hospitalization in non‐dual or dual‐eligible enrollees. Offering any expanded PHR was negatively associated with the probability of a re‐admission for non‐dual enrollees, but the event study plots (A3 of Figure 1) reveal a potential violation of the parallel trends assumption: the pre‐treatment difference was 1.42 percentage points (95% CI: 0.56, 2.29). This increasing pre‐treatment trend in benefit‐offering plans suggests that the DiD estimate may underestimate the true effect of benefit offering on reducing re‐admissions. However, the estimated effect on re‐admissions among non‐dual enrollees should be carefully interpreted given the differential pre‐trends and we do not consider it a causal effect.

The second top panel of Table 2 shows that offering ≥ 2 expanded PHR benefits reduced the annual probability of ED use and hospitalizations among non‐dual eligible enrollees. The estimated effect on ED use was small: a reduction of 0.67 percentage points (95% CI: −1.11, −0.23). The effect on hospitalizations was a 0.69 percentage point decrease (95% CI: −0.96, −0.43). The event‐study plots for these two analyses (A1 and A2 of Figure 2) support the parallel trends assumption, with pre‐treatment differences of 0.12 percentage points for ED use (95% CI: −0.24, 0.48) and 0.12 percentage points for hospitalizations (95% CI: −0.12, 0.37). For dual‐eligible enrollees, offering ≥ 2 expanded PHR benefits reduced the probability of being hospitalized by 0.84 percentage points (95% CI: −1.44, −0.23) with the parallel pre‐treatment trends (B2 of Figure 2), but did not affect ED use or re‐admissions.

The lower panels of Table 2 show that offering SSBCI or offering ≥ 2 SSBCI did not generally reduce acute care utilization for all non‐dual or all dual‐eligible enrollees. We observed only one significant estimate: Offering any SSBCI was associated with a 3.26 percentage point increase in the probability of a re‐admission among dual‐eligible enrollees (95% CI: 1.21, 5.31). However, this counter‐intuitive finding appears to come from differential trends in the outcome between benefit‐offering and not‐offering plans. The event‐study plots (B3 of Appendix Figure A2) reveal positive pre‐treatment trends in re‐admissions for dual‐eligible enrollees (pre‐treatment difference: 2.06 percentage points, 95% CI: 0.03, 3.82). Given this differential pre‐treatment trend, we do not interpret the DiD estimate for re‐admissions among dual enrollees as a causal effect.

### 3.1 Robustness to Deviations From Parallel Trends

We examined the robustness of the statistically significant DiD estimates in Table 2 to deviations from parallel trends in cases where differential pre‐treatment trends were present. Appendix Figure A4 depicts plots from *HonestDiD* for offering any expanded PHR and re‐admissions for non‐dual enrollees, where there were differential pre‐treatment trends (A3 of Figure 1). Figure A4 shows that allowing up to 50% of the pre‐treatment period deviation led to 95% CIs that include zero. This suggests that the DiD estimate on re‐admissions for non‐dual enrollees should not be interpreted as a causal effect. Similarly, we observed a deviation from parallel for the analysis of the effect of offering any SSBCI on re‐admissions among dual‐eligible enrollees, and this analysis produced a counter‐intuitive DiD estimate. However, *HonestDiD* (Appendix Figure A5) indicates that the 95% CIs included zero when a 50% deviation from the pre‐treatment trend was allowed. Thus, the counter‐intuitive, positive DiD effect on re‐admissions for dual‐eligible enrollees should not be interpreted as causal.

### 3.2 Results From Analyses of Highly Frail Enrollees

Turning to high‐frailty groups, enrollee characteristics in the matched sample were generally similar between benefit‐offering and not‐offering plans (Appendix Tables A4.1 through A5.2). Any residual differences in observed characteristics after matching are controlled for in DiD regressions.

Table 3 presents the DiD estimates from analyses of highly frail enrollees. Appendix Figures A6–A9 show corresponding event‐study plots.

**TABLE 3:** Difference‐in‐differences estimates MA's expanded supplemental benefits among highly frail enrollees.

	Non‐dual eligible enrollees			Dual eligible enrollees		
	*N*	Baseline mean (%)	Estimate (95% CI), % point	*N*	Baseline mean (%)	Estimate (95% CI), % point
Offering any expanded PHR benefit						
Prob. of ED use^a^	1,054,628	48.3	−4.05 (−4.43, −3.67)***	249,333	68.8	−3.98 (−4.66, −3.30)***
Prob. of hospitalization^a^	1,054,628	26.5	−2.20 (−2.49, −1.91)***	249,333	38.1	−2.63 (−3.28, −1.99)***
Prob. of re‐admission^a^	32,765	26.3	−2.82 (−4.78, −0.86)*	15,005	32.0	−0.66 (−3.63, 2.31)
Offering ≥ 2 expanded PHR benefits						
Prob. of ED use	326,003	47.4	−1.79 (−2.61, −0.98)***	71,917	65.9	−0.83 (−2.29, 0.64)
Prob. of hospitalization	326,003	25.8	−1.76 (−2.38, −1.14)***	71,917	36.9	−2.05 (−3.43, −0.67)**
Prob. of re‐admission	9976	26.0	−2.09 (−5.77, 1.59)	4173	34.0	−4.61 (−10.56, 1.34)
Offering any SSBCI						
Prob. of ED use	245,400	54.8	−4.21 (−5.12, −3.31)***	154,322	70.7	−3.68 (−4.66, −2.70)***
Prob. of hospitalization	245,400	33.0	−2.75 (−3.53, −1.97)***	154,322	42.3	−1.27 (−2.20, −0.34)**
Prob. of re‐admission	12,362	32.1	−0.03 (−3.88, 3.81)	11,764	31.6	1.97 (−1.97, 5.92)
Offering ≥ 2 SSBCI						
Prob. of ED use	133,733	55.4	**−3.51 (−4.96, −2.05)** ***	84,544	70.6	−1.26 (−2.57, 0.06)
Prob. of hospitalization	133,733	33.4	**−2.05 (−3.12, −0.99)** ***	84,544	42.4	−0.25 (−1.47, 0.97)
Prob. of re‐admission	5708	27.3	−4.18 (−10.62, 2.26)	6089	32.5	2.28 (−2.86, 7.41)

*Note:* Estimates of the overall treatment effects obtained from staggered difference‐in‐differences models using the matched sample, controlling for plan‐county fixed effects, year dummies, enrollee demographics and health risks, area socio‐economic factors and health resource variables, and time‐varying plan attributes, including supplemental benefits other than the benefit in question. Standard errors are clustered at the plan level. Bolded results are statistically significant and supported by parallel trends during the pre‐treatment period.

Abbreviations: %points, percentage points; CI, confidence interval; ED, emergency department; MA, Medicare Advantage; PHR, primarily health‐related; SSBCI, special supplemental benefits for the chronically ill.

^a^ Annual probability of having an ED visit, hospitalization, or re‐admission.

***p‐value < 0.001, **p‐value < 0.01, and *p‐value < 0.05.

These analyses yielded many more significant estimates with expected signs than the analyses of all enrollees, particularly among highly frail non‐dual enrollees (Table 3). Offering any expanded PHR reduced the probability of a re‐admission by −2.82 percentage points (95% CI: −4.78, −0.86), offering ≥ 2 expanded PHR benefits reduced the probability of an ED visit by −1.79 percentage points (95% CI: −2.61, −0.98), and the probability of being hospitalized by −1.76 percentage points (95% CI: −2.38, −1.14), and offering ≥ 2 SSBCI reduced ED use by −3.51 percentage points (95% CI: −4.96, −2.05) and the probability of being hospitalized by −2.05 percentage points (95% CI: −3.12, −0.99). Event‐study plots for these outcomes supported parallel pre‐treatment trends (Appendix Figures A6–A9). These findings suggest that MA plans' provision of expanded supplemental benefits reduces acute care utilization for highly frail non‐dual enrollees.

Offering any expanded PHR benefit also showed significant impacts on ED use and hospitalizations for highly frail non‐dual enrollees, but we observed differential pre‐treatment trends for these analyses (Appendix Figures A6.A1 and A6.A2). The results from *HonestDiD* (Appendix Figure A10) suggest that the DiD estimates are relatively robust to deviations from parallel trends–i.e., 95% CIs did not include zero, even allowing deviations up to the same magnitude as the pre‐treatment difference. However, these effects should be interpreted with caution given the differential pre‐treatment trends. Similarly, offering SSBCI showed significant negative impacts on ED use and hospitalizations, but we observed differential pre‐treatment trends (Appendix Figures A9.A1 and A9.A2) although 95% CIs did not include zero when deviations similar to the pre‐offering trends were allowed (Appendix Figure A11).

For highly frail dual‐eligible enrollees, offering ≥ 2 expanded PHR reduced the probability of being hospitalized, and offering any SSBCI also reduced the probabilities of using ED and being hospitalized. These three analyses had parallel pre‐treatment trends (Appendix Figures A7 and A8). Offering any expanded PHR benefit also reduced ED use and hospitalizations. However, these two analyses had differential pre‐treatment trends (Appendix Figure A6) and 95% CIs that included zero when deviations of about 50% from parallel trends were allowed (Appendix Figure A12).

### 3.3 Results From Secondary Analyses

Appendix Tables A6.1 and A6.2 report the DiD estimates from the analyses of a single benefit category. Consistent with the primary analysis, we observed several significant estimates that are supported by parallel pre‐trends for highly frail non‐dual enrollees: Offering in‐home support services reduced ED use, offering food security benefits reduced hospitalizations and re‐admissions, and offering housing quality benefits reduced ED use and hospitalizations.

## 4 Discussion

Analyzing data from the initial 4 years of MA's expanded supplemental benefits, we found that offering those benefits did not generally reduce enrollees' ED use, hospitalizations, and re‐admissions in all non‐dual or all dual‐eligible enrollees. A few analyses yielded significant results, but their estimates were relatively small or were not robust to potential deviations from parallel trends.

These estimates may be insignificant because we examined the impact of benefit provision, not benefit use. This approach estimates benefit impacts among potential benefit users and likely attenuates the impacts if only a small portion of plan enrollees used expanded supplemental benefits. A survey of about 3000 MA enrollees found that about 12% received a grocery allowance (a food/produce benefit) [34]. However, actual uptake of the benefit among enrollees eligible for SSBCI will likely be higher. The degree of nationwide uptake of expanded supplemental benefits is unknown. We note that our insignificant findings do not imply that MA's expanded supplemental benefits did not reduce acute care utilization for benefit users.

Acute care utilization declined significantly for highly frail non‐dual enrollees in plans offering expanded supplemental benefits, particularly in plans offering multiple benefits. These significant estimates were based on parallel pre‐treatment trends and/or were robust to potential deviations from parallel trends. We believe that highly frail enrollees have a high likelihood of using expanded supplemental benefits, but their actual uptake of benefits is not known. Thus, we do not claim that these estimates are effects of benefit receipt, but they do suggest that MA plans' provision of expanded supplemental benefits may have impacts for certain groups of enrollees, such as high‐risk non‐dual patients.

We observed a few significant estimates from the analyses of highly frail dual‐eligible enrollees. This suggests that offering expanded supplemental benefits might reduce acute care utilization also for dual‐eligible enrollees, but to smaller degrees than for non‐dual enrollees. One possible explanation for this difference is that dual‐eligible enrollees are eligible for Medicaid benefits that are similar to MA's expanded supplemental benefits. Some dual‐eligible enrollees in control plans may receive comparable supports through Medicaid, and MA's expanded supplemental benefits may add little marginal value for dual‐eligible enrollees in treatment plans.

We used a quasi‐experimental study design and analyzed data from enrollees whose plan choice was pre‐determined prior to plans' decisions about benefit offering. This helps isolate the impacts of benefit offering from other factors. However, unobserved health risks may remain that are associated with both continued enrollment in benefit‐offering plans and acute care use. Event‐study estimates from several analyses indicated positive differential trends in acute care utilization between benefit offering and not‐offering plans, suggesting that acute care use might have increased in benefit‐offering plans, even without offering expanded supplemental benefits. Thus, our findings may be conservative estimates of the impact of benefit provision.

Previous studies evaluating interventions to address health‐related social needs showed generally positive findings, such as reducing adverse acute care utilization, although their results are not entirely consistent [4, 35, 36]. The interventions examined in prior literature are similar to the expanded supplemental benefits offered by MA plans. However, our study setting differs from prior interventions, which were often implemented in small settings by local community organizations. MA's expanded supplemental benefits are a large federal initiative to incorporate non‐medical benefits into the health care system through private health insurers.

To our knowledge, we are the first to use nationwide data to examine how this MA initiative affects acute care utilization. Analyzing benefit‐offering impacts among potential users, we found that MA plans' provision of expanded supplemental benefits reduced adverse health events for certain enrollee groups. This effect may be partially through an indirect pathway that expanded benefits free up resources for care management services. A study of one MA insurer showed enrollees who received a grocery card—one of SSBCI—had more outpatient visits, including annual wellness visits, than those who did not [37]. While these findings are positive, continued efforts to assess the impacts of MA's supplemental benefits are warranted, particularly given the growing concern that MA plans use those benefits as a marketing tool to obtain a competitive advantage in the market [38].

Further studies that examine the effects of enrollees' actual use of expanded supplemental benefits would help assess whether continued expansion of MA supplemental benefits should be promoted, or other approaches should be explored to meet enrollees' needs [39, 40]. They could also help refine MA supplemental benefit policy [1]. Evidence on the use of MA's supplemental benefits is scarce because reliable nationwide data on enrollees' benefit use are not yet available. As concerns are raised about the effectiveness of MA supplemental benefits, CMS recently strengthened the requirement for MA plans to report enrollees' use of supplemental benefits, starting in 2024. This is a positive development, and other procedures and regulations to monitor and ensure high compliance with data reporting are also in place [41, 42, 43]. Future research using this new data would provide further evidence on the value of MA's supplemental benefits and help inform policy making.

This study has several limitations. First, we examined the effects of benefit *offering* rather than individuals' benefit *use*. This approach may have attenuated the benefit impacts. The extent of benefit provision was also limited to offering at least two benefits. Second, we focused on the early years of expanded supplemental benefits. MA plans may have refined implementation of expanded supplemental benefits over time, such as identifying enrollees who would most benefit from the benefits. Thus, effects in later years may differ from our estimates. Third, the study outcomes were limited to acute care utilization captured in administrative data. We did not examine changes in unmet social needs, which are important potential outcomes of expanded supplemental benefits, because they are not observable in encounter data. Fourth, despite the quasi‐experimental study design, unobserved risk factors may remain that are correlated with both acute care use and enrollment in benefit‐offering plans (e.g., high‐risk enrollees may tend to stay in a benefit‐offering plan).

Despite these limitations, our study provides the first indication that MA plans' provision of expanded supplemental benefits may reduce acute care use for certain enrollee groups. As spending on MA supplemental benefits rises, continued efforts to evaluate their impacts on diverse outcomes and for benefit users are needed.

## Funding

This work was supported by the National Institute on Aging, 1R01AG069352, 1R01AG099292, P30 AG066587.

## Conflicts of Interest

The authors declare no conflicts of interest.

## Supporting information


**Appendix A1.** Chronic conditions used for the study.
**Appendix A2**. Requirement for plan enrollment and propensity score matching.
**Appendix Table A1**. Steps to identify the study sample and associated numbers.
**Appendix Table A2.1**. Descriptive statistics of study variables for the analyses of expanded PHR benefits: Non‐dual eligible group.
**Appendix Table A2.2**. Descriptive statistics of study variables for the analyses of expanded PHR benefits: Dual‐eligible group.
**Appendix Table A3.1**. Descriptive statistics of study variables for the analyses of SSBCI: Non‐dual eligible group.
**Appendix Table A3.2**. Descriptive statistics of study variables for the analyses of SSBCI: Dual eligible group.
**Appendix Table A4.1**. Descriptive statistics of study variables for the analyses of expanded PHR benefits among highly frail enrollees: Non‐dual eligible group.
**Appendix Table A4.2**. Descriptive statistics of study variables for the analyses of expanded PHR benefits among highly frail enrollees: Dual eligible group.
**Appendix Table A5.1**. Descriptive statistics of study variables for the analyses of SSBCI among highly frail enrollees: Non‐dual eligible group.
**Appendix Table A5.2**. Descriptive statistics of study variables for the analyses of SSBCI among highly frail enrollees: Dual eligible group.
**Appendix Table A6.1**. Difference‐in‐differences estimates from benefit‐specific analyses.
**Appendix Table A6.2**. Difference‐in‐differences estimates from benefit‐specific analyses among highly frail enrollees.
**Appendix Figure A1.1**. Growth in the adoption of expanded supplemental benefits over the study period (2017–2022).
**Appendix Figure A1.2**. Growth in the adoption of a single benefit or benefit group over the study period (2017–2022).
**Appendix Figure A2**. Event‐study plots from the analysis of offering any SSBCI.
**Appendix Figure A3**. Event‐study plots from the analysis of offering ≥ 2 SSBCI.
**Appendix Figure A4**. *HonestDiD* results for the effect of offering any expanded PHR benefit on the annual probability of a re‐admission for non‐dual enrollees.
**Appendix Figure A5**. *HonestDiD* results for the effect of offering any SSBCI on the annual probability of a re‐admission for dual‐eligible enrollees.
**Appendix Figure A6**. Event‐study plots from the analysis of any expanded PHR benefit among highly frail enrollees.
**Appendix Figure A7**. Event‐study plots from the analysis of ≥ 2 expanded PHR benefits among highly frail enrollees.
**Appendix Figure A8**. Event‐study plots from the analysis of any SSBCI among highly frail enrollees.
**Appendix Figure A9**. Event‐study plots from the analysis of ≥ 2 SSBCI among highly frail enrollees.
**Appendix Figure A10**. *HonestDiD* results for highly frail non‐dual enrollees: Analysis of offering any expanded PHR benefit.
**Appendix Figure A11**. *HonestDiD* results for highly frail non‐dual enrollees: Analysis of offering any SSBCI.
**Appendix Figure A12**. *HonestDiD* results for highly frail dual‐eligible enrollees: Analysis of offering any expanded PHR benefit.

## Data Availability

The data supporting the findings of this study were obtained from the Centers for Medicare & Medicaid Services (CMS) under a Data Use Agreement (DUA) and cannot be shared due to the restrictions of that agreement.
