# Supplementary material for: Medicare Advantage's Provision of Expanded Supplemental Benefits and Acute Care Utilization
Source: Health Serv Res. 2026 May 29;61(3):e70136. doi: 10.1111/1475-6773.70136 (PMC13240104; doi:10.1111/1475-6773.70136)
Supplement: Supplementary file 1 — Appendix A1. Chronic conditions used for the study. Appendix A2. Requirement for plan enrollment and propensity score matching. Appendix Table A1. Steps to identify the study sample and associated numbers. Appendix Table A2.1. Descriptive statistics of study variables for the analyses of expanded PHR benefits: Non‐dual eligible group. Appendix Table A2.2. Descriptive statistics of study variables for the analyses of expanded PHR benefits: Dual‐eligible group. Appendix Table A3.1. Descriptive statistics of study variables for the analyses of SSBCI: Non‐dual eligible group. Appendix Table A3.2. Descriptive statistics of study variables for the analyses of SSBCI: Dual eligible group. Appendix Table A4.1. Descriptive statistics of study variables for the analyses of expanded PHR benefits among highly frail enrollees: Non‐dual eligible group. Appendix Table A4.2. Descriptive statistics of study variables for the analyses of expanded PHR benefits among highly frail enrollees: Dual eligible group. Appendix Table A5.1. Descriptive statistics of study variables for the analyses of SSBCI among highly frail enrollees: Non‐dual eligible group. Appendix Table A5.2. Descriptive statistics of study variables for the analyses of SSBCI among highly frail enrollees: Dual eligible group. Appendix Table A6.1. Difference‐in‐differences estimates from benefit‐specific analyses. Appendix Table A6.2. Difference‐in‐differences estimates from benefit‐specific analyses among highly frail enrollees. Appendix Figure A1.1. Growth in the adoption of expanded supplemental benefits over the study period (2017–2022). Appendix Figure A1.2. Growth in the adoption of a single benefit or benefit group over the study period (2017–2022). Appendix Figure A2. Event‐study plots from the analysis of offering any SSBCI. Appendix Figure A3. Event‐study plots from the analysis of offering ≥ 2 SSBCI. Appendix Figure A4. HonestDiD results for the effect of offering any expanded PHR benefit on the annual probabilit [file HESR-61-e70136-s001.docx]

**Supplement to**

Jeah Jung, Ge Song, Roger Feldman, Caroline Carlin, Daniel Polsky, Hyunjee Kim, “Medicare Advantage’s Expanded Supplemental Benefits

and Acute Care Utilization,” *Health Services Research*, 2026

**Table of Contents**

[Appendix A1. Chronic conditions used for the study 4](#_Toc227078602)

[Appendix A2. Requirement for plan enrollment and propensity score matching 5](#_Toc227078603)

[Appendix Table A1. Steps to identify the study sample and associated numbers 7](#_Toc227078604)

[Appendix Table A2.1. Descriptive statistics of study variables for the analyses of expanded PHR benefits: Non-dual eligible group 10](#_Toc227078605)

[Appendix Table A2.2. Descriptive statistics of study variables for the analyses of expanded PHR benefits: Dual-eligible group 12](#_Toc227078606)

[Appendix Table A3.1. Descriptive statistics of study variables for the analyses of SSBCI: Non-dual eligible group 14](#_Toc227078607)

[Appendix Table A3.2. Descriptive statistics of study variables for the analyses of SSBCI: Dual eligible group 16](#_Toc227078608)

[Appendix Table A4.1. Descriptive statistics of study variables for the analyses of expanded PHR benefits among highly frail enrollees: Non-dual eligible group 18](#_Toc227078609)

[Appendix Table A4.2. Descriptive statistics of study variables for the analyses of expanded PHR benefits among highly frail enrollees: Dual eligible group 20](#_Toc227078610)

[Appendix Table A5.1. Descriptive statistics of study variables for the analyses of SSBCI among highly frail enrollees: Non-dual eligible group 22](#_Toc227078611)

[Appendix Table A5.2. Descriptive statistics of study variables for the analyses of SSBCI among highly frail enrollees: Dual eligible group 24](#_Toc227078612)

[Appendix Table A6.1. Difference-in-differences estimates from benefit-specific analyses 26](#_Toc227078613)

[Appendix Table A6.2. Difference-in-differences estimates from benefit-specific analyses among highly frail enrollees 27](#_Toc227078614)

[Appendix Figure A1.1 Growth in the adoption of expanded supplemental benefits over the study period (2017-2022) 28](#_Toc227078615)

[Appendix Figure A1.2 Growth in the adoption of a single benefit or benefit group over the study period (2017-2022) 29](#_Toc227078616)

[Appendix Figure A2. Event-study plots from the analysis of offering any SSBCI 30](#_Toc227078617)

[Appendix Figure A3. Event-study plots from the analysis of offering ≥2 SSBCI 31](#_Toc227078618)

[Appendix Figure A4. *HonestDiD* results for the effect of offering any expanded PHR benefit on the annual probability of a re-admission for non-dual enrollees 32](#_Toc227078619)

[Appendix Figure A5. *HonestDiD* results for the effect of offering any SSBCI on the annual probability of a re-admission for dual-eligible enrollees 33](#_Toc227078620)

[Appendix Figure A6. Event-study plots from the analysis of any expanded PHR benefit among highly frail enrollees 34](#_Toc227078621)

[Appendix Figure A7. Event-study plots from the analysis of ≥ 2 expanded PHR benefits among highly frail enrollees 35](#_Toc227078622)

[Appendix Figure A8. Event-study plots from the analysis of any SSBCI among highly frail enrollees 36](#_Toc227078623)

[Appendix Figure A9. Event-study plots from the analysis of ≥ 2 SSBCI among highly frail enrollees 37](#_Toc227078624)

[Appendix Figure A10. *HonestDiD* results for highly frail non-dual enrollees: Analysis of offering any expanded PHR benefit 38](#_Toc227078625)

[Appendix Figure A11. *HonestDiD* results for highly frail non-dual enrollees: Analysis of offering any SSBCI 39](#_Toc227078626)

[Appendix Figure A12. *HonestDiD* results for highly frail dual-eligible enrollees: Analysis of offering any expanded PHR benefit 40](#_Toc227078627)

# Appendix A1. Chronic conditions used for the study

- Autoimmune diseases: Arthritis - rheumatoid arthritis or osteoarthritis
- Cancer: Breast, colon, endometrial, lung, prostate, or urologic cancer
- Cardiovascular disease: Acute Myocardial Infarction (AMI), ischemic heart disease, atrial fibrillation
- Dementia: Alzheimer's disease, dementia other than Alzheimer’s
- Diabetes
- Heart failure
- Hematologic disorders: Anemia
- Lung diseases: Chronic obstructive pulmonary disease, pneumonia, asthma
- Mental health disorders: Depression
- Neurologic disorders: Parkinson's disease
- Renal diseases: Chronic kidney disease
- Stroke

We used the Chronic Condition Warehouse (CCW) algorithm^1^ to construct chronic condition indicators. The CCW algorithm pools two years of data for all but three conditions above. We pooled two years of data from inpatient and outpatient services, regardless of enrollees’ coverage – i.e., we used both traditional Medicare (TM) claims and Medicare Advantage (MA) encounter data if enrollees were in MA in a given year but were in TM in the prior year. This helps minimize potential measurement errors due to incomplete MA encounter records. The remaining three conditions are AMI, stroke, and pneumonia, which are all acute and not persistent. It would not be reasonable to pool the data for these conditions.

Reference:

1. Chronic Condition Warehouse (CCW). Chronic Conditions. Chronic Conditions Data Warehouse. Accessed April 1, 2026. https://www2.ccwdata.org/condition-categories-chronic

# Appendix A2. Requirement for plan enrollment and propensity score matching

To address enrollees’ non-random plan choice (i.e., certain patients selectively choosing a benefit-offering plan), we imposed a requirement for plan enrollment: enrollees must stay in the same plan during the pre- and post-offering periods as long as they are observed in the sample. We did not require continuous enrollment in the same plan over the full study period because it would be overly restrictive and may identify a selective sample, limiting the study’s generalizability. Enrollees were allowed to exit the sample: for example, enrollees who switched to traditional Medicare during the follow-up period exited the sample. The requirement of staying in the same plan identifies enrollees in the treatment group who chose their plan prior to the plan’s benefit offering, and enrollees in the control group who chose their plan prior to the policy change that allowed plans to offer an expanded supplemental benefit.

To further ensure comparability between treatment and control groups, we used propensity score matching based on characteristics measured one year prior to benefit offering. Matching was conducted separately for each expanded supplemental benefit or benefit group and within relevant enrollee subgroups. Because of the staggered adoption of expanded supplemental benefits by MA plans, we divided the treatment group into several cohorts depending on the year of benefit adoption and performed enrollee matching separately for each cohort. We pooled all matched cohorts together to create the analytic sample for the benefit in question. The matching procedure is illustrated below, using the sample for the analysis of offering any expanded primarily health-related (PHR) benefit as an example.

Plans gradually adopted expanded PHR benefits beginning in 2019. We first identified the year that each plan adopted any expanded PHR. For enrollees in the 2019 adoption cohort, we used contemporaneous (2019) observations from plans that never adopted an expanded PHR throughout the study period as the control group. We then estimated a logit model predicting enrollment in a treatment plan as a function of patient-, area-, and plan-level covariates (all control variables used in the study). Using the estimated propensity scores, we implemented 1:1 nearest-neighbor matching within a caliper set at 10% of the standard deviation of the propensity score, without replacement. This matching procedure was conducted separately for non-dual-eligible and dual-eligible enrollees. We repeated the same process for other adoption cohorts (2020, 2021, etc.). We pooled all the matched cohorts to construct the analytic sample used in subsequent analyses.

The pooled matched sample consisted of 1,837,571 non-dual and 455,186 dual enrollee-years in benefit-offering plans (the treatment group) and 1,832,329 non-dual and 363,152 dual enrollee-years in non-offering plans (the control group). The number of observations for the treatment and control groups are not identical because 1:1 matching was performed at baseline only. Matched observations may contribute differing numbers of follow-up periods before and after the baseline year, resulting in different group sizes in the longitudinal analytic sample. We applied this process separately to each benefit indicator used for the study. We confirmed that all matched observations were on the common support.

Appendix Tables A2.1 through A5.2 report enrollee characteristics of the study sample before and after matching, separately for the sample used for each analysis. To assess the matching performance, we obtained standardized differences for each variable between treatment and control groups for both unmatched and matched samples. Standardized differences substantially decreased in the matched samples, compared with the unmatched samples. They were lower than 10% for most variables in all matched samples. One exception was enrollment in integrated D-SNPs, which was slightly higher in the treatment group than in the control group for the analysis of offering SSBCI among dual-eligible enrollees (29% vs. 25%; standardized difference = 10.5%; Appendix Table A5.2). Any residual differences in observed characteristics are controlled for in regressions.

As an example, we compare the distributions of enrollee characteristics between unmatched and matched samples using Appendix Tables A2.1 and A2.2 (the sample used for the analysis of offering any expanded Primarily Health Related [PHR] benefit). Before matching, enrollees in benefit-offering plans were more likely to be Hispanic, compared with enrollees in not-offering plans. The mean frailty score was lower in benefit-offering plans than in not-offering plans (0.15 vs. 0.16 for non-dual, and 0.18 vs. 0.19 for dual-eligible enrollees). Among dual-eligible individuals, enrollment in coordination-only D-SNPs was higher in benefit-offering plans than in not-offering plans. However, after matching, enrollee characteristics between benefit-offering and not-offering plans were similar. The mean frailty score was the same between benefit-offering and not-offering plans. The difference in the prevalence of chronic conditions was < 1 percentage point.

# Appendix Table A1. Steps to identify the study sample and associated numbers

| **Panel A: Analyses of Expanded PHR benefits** | | | | | | |
| --- | --- | --- | --- | --- | --- | --- |
|  | Non-dual eligible group  (enrollee-years) | | | Dual eligible group  (enrollee-years) | | |
|  | N | Excluded | Remaining | N | Excluded | Remaining |
| Random 20% sample of MA enrollees from 2017 to 2022 national MA encounter data^a^ | 21,543,121 |  | 21,543,121 | 5,604,155 |  | 5,604,155 |
| Excluded enrollees in C-SNPs | 21,543,121 | 325,861 | 21,217,260 | 5,604,155 | 218,797 | 5,385,358 |
| Excluded enrollees with End-Stage Renal Disease (ESRD) | 21,217,260 | 2,982 | 21,214,278 | 5,385,358 | 4,846 | 5,380,512 |
| Limited to enrollees in MA contracts with high data completeness | 21,214,278 | 1,054,143 | 20,160,135 | 5,380,512 | 256,402 | 5,124,110 |
| Excluded enrollees in treatment plans during the years after dropping the benefit | 20,160,135 | 1,602,396 | 18,557,739 | 5,124,110 | 572,235 | 4,551,875 |
| Limited to treatment enrollees in plans with a pre-adoption period | 18,557,739 | 344,818 | 18,212,921 | 4,551,875 | 160,368 | 4,391,507 |
| Excluded enrollees who switched between control and treatment plans across years | 18,212,921 | 2,816,075 | 15,396,846 | 4,391,507 | 1,122,712 | 3,268,795 |
| Limited the treatment group to enrollees in the same plan pre- and post-benefit adoption periods | 15,396,846 | 1,078,534 | 14,318,312 | 3,268,795 | 601,574 | 2,667,221 |
| Limited the control group to enrollees in the same plan before and after policy benefit adoption years | 14,318,312 | 4,280,120 | 10,038,192 | 2,667,221 | 937,462 | 1,729,759 |
| Excluded long-term (>100 days) nursing home stayers | 10,038,192 | 24,735 | 10,013,457 | 1,729,759 | 77,857 | 1,651,902 |
| Excluded records with missing covariates | 10,013,457 | 189,380 | 9,824,077 | 1,651,902 | 36,711 | 1,615,191 |
| Unmatched sample that used for matching | 9,824,077 |  |  | 1,615,191 |  |  |
| Treatment group | 1,878,404 |  |  | 483,711 |  |  |
| Control group | 7,945,673 |  |  | 1,131,480 |  |  |
| Matched sample^b^ | 3,669,900 |  |  | 818,338 |  |  |
| Treatment group | 1,837,571 |  |  | 455,186 |  |  |
| Control group | 1,832,329 |  |  | 363,152 |  |  |
| **Panel B: Analyses of SSBCI** | | | | | | |
|  | Non-dual eligible group  (enrollee-years) | | | Dual eligible group  (enrollee-years) | | |
|  | N | Excluded | N | Excluded | N | Excluded |
| Random 20% sample of MA enrollees from 2017 to 2022 national MA encounter data^a^ | 21,543,121 |  | 21,543,121 | 5,604,155 |  | 5,604,155 |
| Excluded enrollees in C-SNPs | 21,543,121 | 325,861 | 21,217,260 | 5,604,155 | 218,797 | 5,385,358 |
| Excluded enrollees with End-Stage Renal Disease (ESRD) | 21,217,260 | 2,982 | 21,214,278 | 5,385,358 | 4,846 | 5,380,512 |
| Limited to enrollees in MA contracts with high data completeness | 21,214,278 | 1,054,143 | 20,160,135 | 5,380,512 | 256,402 | 5,124,110 |
| Excluded enrollees in treatment plans during the years after dropping the benefit | 20,160,135 | 92,576 | 20,067,559 | 5,124,110 | 21,952 | 5,102,158 |
| Limited to treatment enrollees in plans with a pre-adoption period | 20,067,559 | 192,258 | 19,875,301 | 5,102,158 | 106,427 | 4,995,731 |
| Excluded enrollees who switched between control and treatment plans across years | 19,875,301 | 1,384,784 | 18,490,517 | 4,995,731 | 1,058,177 | 3,937,554 |
| Limited the treatment group to enrollees in the same plan pre- and post-benefit adoption periods | 18,490,517 | 338,300 | 18,152,217 | 3,937,554 | 321,613 | 3,615,941 |
| Limited the control group to enrollees in the same plan before- and after-policy benefit adoption years | 18,152,217 | 6,188,527 | 11,963,690 | 3,615,941 | 1,408,163 | 2,293,952 |
| Excluded long-term (>100 days) nursing home stayers | 11,963,690 | 27,166 | 11,936,524 | 2,293,952 | 90,287 | 2,117,491 |
| Limited to enrollees with a chronic condition(s) | 11,936,524 | 3,205,323 | 8,731,201 | 2,117,491 | 380,252 | 1,737,239 |
| Excluded records with missing covariates | 8,731,201 | 169,991 | 8,561,210 | 1,737,239 | 39,167 | 1,698,072 |
| Unmatched sample that used for matching | 8,561,210 |  |  | 1,698,072 |  |  |
| Treatment group | 528,812 |  |  | 353,891 |  |  |
| Control group | 8,032,398 |  |  | 1,344,181 |  |  |
| Matched sample^b^ | 801,604 |  |  | 489,663 |  |  |
| Treatment group | 409,608 |  |  | 283,223 |  |  |
| Control group | 391,996 |  |  | 206,440 |  |  |

Abbreviations: PHR, primarily health-related; SSBCI, special supplemental benefits for the chronically Ill; C-SNPs, chronic condition special needs plans

^a^ Enrollees must have continuous enrollment in MA with Parts A and B coverage in a given year and reside in 50 states or D.C. Enrollees in special plans (employer-sponsored, cost, PACE, or demonstration plans) are excluded.

^b^ Matched sample uses 1:1 nearest-neighbor propensity score matching with a caliper of 10% of the standard deviation of the propensity score without replacement on baseline characteristics, measured one year before benefit offering. Matching is conducted separately by benefit and adoption-year cohorts due to staggered implementation, then all matched cohorts are pooled to create the final matched sample for each benefit.

# Appendix Table A2.1. Descriptive statistics of study variables for the analyses of expanded PHR benefits: Non-dual eligible group

|  | Non-dual eligible group, Mean (SD) or % | | | | | | | | |  |  |
| --- | --- | --- | --- | --- | --- | --- | --- | --- | --- | --- | --- |
|  | Before matching | | | | After matching | | | | | | |
|  | Benefit offering plans | Not-offering plans | Standardized difference | | Benefit offering plans | | Not-offering plans | | Standardized difference | | |
| Female | 55.8% | 54.7% | 2.2% | | 55.7% | | 55.7% | | 0.0% | | |
| Age |  |  |  | |  | |  | |  | | |
| Age < 65 | 6.5% | 5.3% | 5.0% | | 6.4% | | 5.9% | | 2.0% | | |
| 65 ≤ Age < 70 | 23.4% | 25.6% | -5.1% | | 23.4% | | 21.3% | | 5.0% | | |
| 70 ≤ Age < 75 | 27.0% | 25.8% | 2.7% | | 27.1% | | 28.2% | | -2.6% | | |
| 75 ≤ Age < 80 | 20.0% | 19.1% | 2.2% | | 20.0% | | 21.2% | | -2.9% | | |
| 80 ≤ Age < 85 | 12.5% | 12.2% | 0.8% | | 12.5% | | 12.9% | | -1.1% | | |
| Age ≥ 85 | 10.8% | 12.1% | -4.1% | | 10.7% | | 10.5% | | 0.6% | | |
| Race |  |  |  | |  | |  | |  | | |
| Asian | 3.7% | 3.3% | 2.2% | | 3.7% | | 3.3% | | 2.1% | | |
| Hispanic | 8.5% | 6.4% | 7.9% | | 8.4% | | 7.9% | | 1.9% | | |
| Non-Hispanic Black | 6.7% | 9.6% | -10.6% | | 6.7% | | 7.3% | | -2.5% | | |
| Non-Hispanic White | 78.4% | 77.8% | 1.3% | | 78.5% | | 78.6% | | -0.4% | | |
| Other race^a^ | 2.8% | 2.9% | -0.6% | | 2.8% | | 2.9% | | -0.6% | | |
| Frailty score, Mean (SD) | 0.15 (0.06) | 0.16 (0.06) | -11.4% | | 0.15 (0.06) | | 0.15 (0.06) | | -1.6% | | |
| Chronic Conditions^b^ |  |  |  | |  | |  | |  | | |
| Anemia | 13.9% | 16.8% | -7.9% | | 13.9% | | 14.7% | | -2.1% | | |
| Arthritis^c^ | 29.6% | 31.8% | -4.8% | | 29.6% | | 30.7% | | -2.5% | | |
| Cancer |  |  |  | |  | |  | |  | | |
| Breast | 3.8% | 4.2% | -2.0% | | 3.8% | | 4.0% | | -0.7% | | |
| Colon | 1.3% | 1.5% | -1.5% | | 1.3% | | 1.3% | | 0.0% | | |
| Endometrial | 0.5% | 0.6% | -0.8% | | 0.5% | | 0.5% | | -0.3% | | |
| Lung | 0.8% | 1.2% | -3.9% | | 0.8% | | 0.8% | | -0.2% | | |
| Prostate | 4.1% | 4.5% | -2.4% | | 4.1% | | 4.2% | | -0.6% | | |
| Urologic | 0.6% | 0.8% | -1.7% | | 0.6% | | 0.6% | | -0.2% | | |
| Cardiovascular disease |  |  |  | |  | |  | |  | | |
| AMI | 0.7% | 1.1% | -4.0% | | 0.7% | | 0.9% | | -1.5% | | |
| Atrial fibrillation | 10.6% | 12.6% | -6.4% | | 10.6% | | 10.7% | | -0.4% | | |
| Ischemic heart disease | 16.8% | 19.1% | -6.2% | | 16.7% | | 17.2% | | -1.2% | | |
| Chronic kidney disease | 19.8% | 19.1% | 1.9% | | 19.7% | | 18.8% | | 2.2% | | |
| Dementia |  |  |  | |  | |  | |  | | |
| Alzheimer's | 1.2% | 1.9% | -5.9% | | 1.2% | | 1.2% | | -0.3% | | |
| Non-Alzheimer's | 3.4% | 5.4% | -9.8% | | 3.4% | | 3.6% | | -0.9% | | |
| Depress | 15.6% | 15.3% | 0.7% | | 15.5% | | 15.1% | | 1.1% | | |
| Diabetes | 25.5% | 27.2% | -3.9% | | 25.5% | | 25.9% | | -1.0% | | |
| Heart failure | 8.9% | 10.5% | -5.2% | | 8.9% | | 8.7% | | 0.8% | | |
| Lung diseases |  |  |  | |  | |  | |  | | |
| Asthma | 5.9% | 6.8% | -3.9% | | 5.8% | | 6.0% | | -0.6% | | |
| COPD | 12.6% | 12.5% | 0.2% | | 12.5% | | 12.0% | | 1.7% | | |
| Pneumonia | 2.5% | 3.9% | -8.1% | | 2.5% | | 2.9% | | -3.0% | | |
| Parkinson's | 1.0% | 1.4% | -3.1% | | 1.0% | | 1.0% | | 0.0% | | |
| Stroke | 3.2% | 3.9% | -3.8% | | 3.2% | | 3.4% | | -1.3% | | |
| County-level health care resources, Mean (SD) | | | |  | |  | |  | | |  |
| Beds/1000, No. | 2.86 (1.77) | 2.86 (1.95) | 0.2% | | 2.86 (1.72) | | 2.85 (1.85) | | 0.9% | | |
| Doctors/1000, No. | 3.60 (2.25) | 3.47 (2.32) | 5.5% | | 3.60 (2.22) | | 3.61 (2.40) | | -0.4% | | |
| SNF beds/1000, No. | 5.14 (2.65) | 5.24 (2.76) | -3.9% | | 5.17 (2.65) | | 5.23 (2.66) | | -2.4% | | |
| Rural | 12.6% | 17.4% | -13.6% | | 12.6% | | 13.1% | | -1.6% | | |
| ZIP code demographics, Mean (SD) | | | | |  | |  | |  | | |
| College educated | 32.50 (15.77) | 32.98 (16.73) | -2.9% | | 32.50 (15.78) | | 33.05 (16.44) | | -3.5% | | |
| Speaking English only | 83.60 (19.10) | 86.35 (17.44) | -15.0% | | 83.61 (19.05) | | 84.69 (18.14) | | -5.8% | | |
| Under federal poverty level | 11.80 (7.17) | 11.98 (7.40) | -2.5% | | 11.80 (7.18) | | 11.68 (7.15) | | 1.8% | | |
| Supplemental benefits |  |  |  | |  | |  | |  | | |
| Medical transportation | 36.4% | 14.1% | 53.1% | | 36.0% | | 20.8% | | 34.2% | | |
| Meals (a limited period) | 35.5% | 28.3% | 15.4% | | 35.0% | | 28.1% | | 14.9% | | |
| Food security | 8.7% | 1.1% | 35.9% | | 8.4% | | 1.2% | | 34.2% | | |
| Housing quality | 3.0% | 0.2% | 22.6% | | 2.9% | | 0.3% | | 20.5% | | |
| Non-medical transportation | 4.6% | 0.1% | 30.4% | | 4.6% | | 0.1% | | 30.6% | | |
| Social needs benefit | 0.5% | 0.3% | 4.5% | | 0.5% | | 0.2% | | 5.4% | | |
| General supports for living | 1.5% | 0.0% | 17.0% | | 1.5% | | 0.0% | | 17.2% | | |
| No. of enrollees | 489,534 | 2,312,069 |  | | 470,146 | | 470,146 | |  | | |
| No. of enrollee-years | 1,878,404 | 7,945,673 |  | | 1,837,571 | | 1,832,329 | |  | | |

Abbreviations: PHR, primarily health-related; SD, standard deviation; AMI, acute myocardial infarction; COPD, chronic obstructive pulmonary disease; SNF, skilled nursing facility.

^a^ Other race is a category defined in the Master Beneficiary Summary File (MBSF).

^b^ Following the Chronic Condition Warehouse algorithm, two years of data are used to construct all conditions except for AMI, pneumonia, and stroke.

^c^ Arthritis includes rheumatoid arthritis and osteoarthritis.

# Appendix Table A2.2. Descriptive statistics of study variables for the analyses of expanded PHR benefits: Dual-eligible group

|  | Dual eligible group, Mean (SD) or % | | | | | | |
| --- | --- | --- | --- | --- | --- | --- | --- |
|  | Before matching | | | After matching | | |  |
|  | Benefit offering plans | Not-offering plans | Standardized difference | Benefit offering plans | Not-offering plans | Standardized difference |  |
| Female | 63.4% | 61.1% | 4.8% | 63.3% | 63.6% | -0.5% |  |
| Age |  |  |  |  |  |  |  |
| Age < 65 | 32.6% | 30.8% | 3.9% | 32.8% | 32.8% | 0.1% |  |
| 65 ≤ Age < 70 | 18.9% | 22.4% | -8.8% | 18.8% | 18.3% | 1.2% |  |
| 70 ≤ Age < 75 | 18.4% | 16.3% | 5.4% | 18.4% | 18.3% | 0.2% |  |
| 75 ≤ Age < 80 | 13.2% | 11.9% | 3.7% | 13.2% | 13.4% | -0.6% |  |
| 80 ≤ Age < 85 | 8.9% | 8.7% | 0.6% | 8.9% | 9.0% | -0.6% |  |
| Age ≥ 85 | 8.1% | 9.9% | -6.1% | 8.0% | 8.2% | -0.7% |  |
| Race |  |  |  |  |  |  |  |
| Asian | 6.3% | 8.4% | -8.1% | 6.4% | 7.2% | -3.2% |  |
| Hispanic | 23.9% | 21.5% | 5.8% | 23.6% | 23.2% | 1.0% |  |
| Non-Hispanic Black | 20.5% | 22.5% | -4.8% | 20.7% | 21.2% | -1.2% |  |
| Non-Hispanic White | 47.3% | 45.0% | 4.6% | 47.3% | 46.3% | 2.1% |  |
| Other race^a^ | 2.0% | 2.7% | -4.4% | 2.0% | 2.2% | -1.3% |  |
| Frailty score, Mean (SD) | 0.18 (0.07) | 0.19 (0.08) | -5.7% | 0.18 (0.07) | 0.18 (0.07) | 0.0% |  |
| Chronic Conditions^b^ |  |  |  |  |  |  |  |
| Anemia | 21.0% | 24.1% | -7.5% | 21.1% | 22.1% | -2.5% |  |
| Arthritis^c^ | 39.4% | 36.7% | 5.7% | 39.4% | 38.7% | 1.6% |  |
| Cancer |  |  |  |  |  |  |  |
| Breast | 3.2% | 3.3% | -1.0% | 3.2% | 3.4% | -1.2% |  |
| Colon | 1.3% | 1.6% | -2.0% | 1.3% | 1.4% | -0.4% |  |
| Endometrial | 0.5% | 0.6% | -0.6% | 0.5% | 0.6% | -0.3% |  |
| Lung | 1.0% | 1.5% | -5.1% | 1.0% | 1.0% | -0.7% |  |
| Prostate | 2.2% | 2.3% | -0.5% | 2.2% | 2.1% | 0.6% |  |
| Urologic | 0.6% | 0.6% | -0.6% | 0.6% | 0.6% | -0.2% |  |
| Cardiovascular disease |  |  |  |  |  |  |  |
| AMI | 1.0% | 1.7% | -6.4% | 1.0% | 1.3% | -2.8% |  |
| Atrial fibrillation | 8.4% | 10.5% | -7.1% | 8.4% | 8.6% | -1.0% |  |
| Ischemic heart disease | 18.3% | 19.7% | -3.5% | 18.4% | 18.3% | 0.3% |  |
| Chronic kidney disease | 24.2% | 22.8% | 3.2% | 24.3% | 22.5% | 4.1% |  |
| Dementia |  |  |  |  |  |  |  |
| Alzheimer's | 1.8% | 2.8% | -7.0% | 1.7% | 1.8% | -0.7% |  |
| Non-Alzheimer's | 5.6% | 8.5% | -11.4% | 5.5% | 5.8% | -1.6% |  |
| Depress | 31.9% | 28.6% | 7.2% | 32.0% | 30.2% | 3.8% |  |
| Diabetes | 38.1% | 38.8% | -1.5% | 38.2% | 38.6% | -0.7% |  |
| Heart failure | 13.4% | 15.4% | -5.5% | 13.4% | 13.5% | -0.1% |  |
| Lung diseases |  |  |  |  |  |  |  |
| Asthma | 11.5% | 12.2% | -2.2% | 11.7% | 12.3% | -1.9% |  |
| COPD | 23.8% | 20.8% | 7.3% | 23.9% | 21.8% | 5.1% |  |
| Pneumonia | 4.4% | 6.6% | -9.6% | 4.3% | 5.1% | -3.6% |  |
| Parkinson's | 1.2% | 1.4% | -2.6% | 1.1% | 1.1% | 0.0% |  |
| Stroke | 4.0% | 5.4% | -6.4% | 4.0% | 4.6% | -2.9% |  |
| Enrollment in D-SNPs |  |  |  |  |  |  |  |
| Co-only D-SNPs | 51.3% | 38.7% | 25.5% | 52.7% | 49.7% | 6.1% |  |
| Integrated D-SNPs^d^ | 17.0% | 15.6% | 3.8% | 16.7% | 15.8% | 2.6% |  |
| County-level health care resources, Mean (SD) | | | |  |  |  |  |
| Beds/1000, No. | 3.15 (1.95) | 3.12 (2.11) | 1.5% | 3.16 (1.85) | 3.13 (2.20) | 1.3% |  |
| Doctors/1000, No. | 3.60 (2.47) | 3.99 (2.94) | -14.6% | 3.64 (2.50) | 3.79 (2.70) | -5.7% |  |
| SNF beds/1000, No. | 5.11 (2.56) | 5.15 (2.66) | -1.6% | 5.14 (2.55) | 5.18 (2.58) | -1.9% |  |
| Rural | 15.9% | 15.0% | 2.4% | 15.4% | 15.5% | -0.4% |  |
| ZIP code demographics, Mean (SD) | | | |  |  |  |  |
| College educated | 25.89 (14.14) | 27.87 (15.39) | -13.4% | 25.97 (14.15) | 26.72 (14.62) | -5.3% |  |
| Speaking English only | 76.91 (25.75) | 80.75 (23.67) | -15.5% | 76.84 (25.63) | 78.03 (24.37) | -4.7% |  |
| Under federal poverty level | 16.99 (9.10) | 16.76 (9.41) | 2.5% | 17.02 (9.10) | 17.11 (9.51) | -0.9% |  |
| Supplemental benefits |  |  |  |  |  |  |  |
| Medical transportation | 77.3% | 57.8% | 42.6% | 77.4% | 70.5% | 16.0% |  |
| Meals (a limited period) | 60.7% | 57.6% | 6.4% | 60.7% | 54.4% | 12.9% |  |
| Food security | 13.4% | 8.8% | 14.5% | 13.3% | 7.3% | 19.9% |  |
| Housing quality | 6.2% | 1.9% | 22.3% | 5.7% | 2.0% | 19.5% |  |
| Non-medical transportation | 8.4% | 0.9% | 36.0% | 8.1% | 0.9% | 35.0% |  |
| Social needs benefit | 1.0% | 1.1% | -1.1% | 1.0% | 0.4% | 6.6% |  |
| General supports for living | 4.4% | 0.7% | 23.4% | 4.3% | 0.4% | 26.0% |  |
| No. of enrollees | 137,992 | 445,932 |  | 122,318 | 122,318 |  |  |
| No. of enrollee-years | 483,711 | 1,131,480 |  | 455,186 | 363,152 |  |  |

Abbreviations: PHR, primarily health-related; SD, standard deviation; AMI, acute myocardial infarction; COPD, chronic obstructive pulmonary disease; SNF, skilled nursing facility; D-SNPs, dual-eligible special needs plans.

^a^ Other race is a category defined in the Master Beneficiary Summary File (MBSF).

^b^ Following the Chronic Condition Warehouse algorithm, two years of data are used to construct all conditions except for AMI, pneumonia, and stroke.

^c^ Arthritis includes rheumatoid arthritis and osteoarthritis.

^d^ Integrated D-SNPs include fully integrated dual-eligible special needs plans (FIDE) or highly integrated dual-eligible special needs plans (HIDE).

# Appendix Table A3.1. Descriptive statistics of study variables for the analyses of SSBCI: Non-dual eligible group

|  | Non-dual eligible group, Mean (SD) or % | | | | | | | |
| --- | --- | --- | --- | --- | --- | --- | --- | --- |
|  | | Before matching | | | After matching | | |  |
|  | | Benefit offering plans | Not-offering plans | Standardized difference | Benefit offering plans | Not-offering plans | Standardized difference |  |
| Female | | 55.1% | 54.2% | 1.8% | 54.4% | 54.4% | 0.0% |  |
| Age | |  |  |  |  |  |  |  |
| Age < 65 | | 7.4% | 6.1% | 5.3% | 7.8% | 8.2% | -1.4% |  |
| 65 ≤ Age < 70 | | 18.3% | 20.3% | -5.0% | 18.0% | 18.8% | -2.1% |  |
| 70 ≤ Age < 75 | | 24.9% | 25.1% | -0.4% | 24.7% | 25.5% | -1.8% |  |
| 75 ≤ Age < 80 | | 21.2% | 20.6% | 1.5% | 20.9% | 21.0% | -0.2% |  |
| 80 ≤ Age < 85 | | 14.5% | 13.9% | 1.6% | 14.6% | 13.9% | 2.0% |  |
| Age ≥ 85 | | 13.7% | 14.1% | -1.1% | 14.0% | 12.7% | 4.0% |  |
| Race | |  |  |  |  |  |  |  |
| Asian | | 3.4% | 3.0% | 2.3% | 3.7% | 3.0% | 3.6% |  |
| Hispanic | | 11.0% | 7.4% | 12.5% | 11.0% | 10.7% | 1.0% |  |
| Non-Hispanic Black | | 12.3% | 9.3% | 9.9% | 11.5% | 11.3% | 0.6% |  |
| Non-Hispanic White | | 71.0% | 77.8% | -15.5% | 71.7% | 72.7% | -2.3% |  |
| Other race^a^ | | 2.3% | 2.6% | -2.0% | 2.2% | 2.3% | -0.7% |  |
| Frailty score, Mean (SD) | | 0.17 (0.06) | 0.17 (0.06) | -7.8% | 0.17 (0.06) | 0.17 (0.06) | -0.5% |  |
| Chronic Conditions^b^ | |  |  |  |  |  |  |  |
| Anemia | | 21.8% | 22.3% | -1.3% | 22.1% | 21.6% | 1.2% |  |
| Arthritis^c^ | | 41.5% | 42.8% | -2.8% | 41.5% | 42.1% | -1.2% |  |
| Cancer | |  |  |  |  |  |  |  |
| Breast | | 5.2% | 5.6% | -1.9% | 5.3% | 5.3% | 0.0% |  |
| Colon | | 1.9% | 2.0% | -0.3% | 2.0% | 1.9% | 0.5% |  |
| Endometrial | | 0.7% | 0.7% | -0.3% | 0.7% | 0.7% | 0.1% |  |
| Lung | | 1.2% | 1.5% | -3.2% | 1.2% | 1.2% | 0.4% |  |
| Prostate | | 5.9% | 6.0% | -0.6% | 5.9% | 5.7% | 0.8% |  |
| Urologic | | 0.8% | 1.0% | -1.7% | 0.9% | 0.9% | 0.4% |  |
| Cardiovascular disease | |  |  |  |  |  |  |  |
| AMI | | 1.1% | 1.4% | -3.2% | 1.1% | 1.2% | -0.9% |  |
| Atrial fibrillation | | 14.8% | 16.7% | -5.1% | 15.6% | 15.5% | 0.4% |  |
| Ischemic heart disease | | 24.4% | 25.5% | -2.6% | 25.8% | 25.6% | 0.5% |  |
| Chronic kidney disease | | 29.0% | 26.6% | 5.4% | 28.9% | 27.6% | 2.7% |  |
| Dementia | |  |  |  |  |  |  |  |
| Alzheimer's | | 1.9% | 2.5% | -4.0% | 1.9% | 1.8% | 1.2% |  |
| Non-Alzheimer's | | 5.3% | 7.0% | -7.3% | 5.4% | 5.3% | 0.4% |  |
| Depress | | 20.8% | 21.5% | -1.6% | 21.1% | 21.8% | -1.8% |  |
| Diabetes | | 37.7% | 36.9% | 1.7% | 39.5% | 40.3% | -1.7% |  |
| Heart failure | | 14.4% | 13.9% | 1.3% | 14.3% | 13.8% | 1.4% |  |
| Lung diseases | |  |  |  |  |  |  |  |
| Asthma | | 7.7% | 8.9% | -4.5% | 8.1% | 8.4% | -1.1% |  |
| COPD | | 17.5% | 17.3% | 0.5% | 17.9% | 17.7% | 0.4% |  |
| Pneumonia | | 3.6% | 5.0% | -7.1% | 3.7% | 4.0% | -1.8% |  |
| Parkinson's | | 1.4% | 1.8% | -3.1% | 1.4% | 1.4% | 0.2% |  |
| Stroke | | 4.8% | 5.1% | -1.8% | 4.8% | 4.7% | 0.7% |  |
| County-level health care resources, Mean (SD) | | | | |  |  |  |  |
| Beds/1000, No. | | 3.01 (2.04) | 2.86 (1.87) | 7.9% | 3.07 (2.20) | 3.04 (2.11) | 1.3% |  |
| Doctors/1000, No. | | 3.77 (2.53) | 3.46 (2.24) | 13.0% | 3.80 (2.69) | 3.66 (2.46) | 5.3% |  |
| SNF beds/1000, No. | | 5.12 (2.43) | 5.25 (2.76) | -4.9% | 5.40 (2.38) | 5.43 (3.07) | -1.1% |  |
| Rural | | 12.0% | 16.2% | -12.1% | 12.8% | 13.9% | -3.1% |  |
| ZIP code demographics, Mean (SD) | | | | |  |  |  |  |
| College educated | | 31.47 (15.85) | 32.40 (16.29) | -5.8% | 31.36 (16.00) | 31.12 (15.90) | 1.5% |  |
| Speaking English only | | 81.56 (22.23) | 85.90 (17.86) | -21.5% | 82.34 (21.64) | 84.19 (19.30) | -9.0% |  |
| Under federal poverty level | | 12.74 (8.00) | 12.06 (7.35) | 8.8% | 12.73 (8.22) | 12.75 (7.64) | -0.3% |  |
| Supplemental benefits | |  |  |  |  |  |  |  |
| Medical transportation | | 50.3% | 18.5% | 71.0% | 48.6% | 37.9% | 21.7% |  |
| Meals (a limited period) | | 54.2% | 29.0% | 52.9% | 50.2% | 47.8% | 4.8% |  |
| Adult day health | | 9.7% | -^d^ | - | 9.5% | - | - |  |
| Home-based palliative care | | 9.3% | 1.7% | 34.0% | 8.7% | 6.6% | 7.8% |  |
| Support for caregivers | | 5.0% | 4.5% | 2.0% | 4.1% | 2.1% | 11.8% |  |
| In-home support | | 18.3% | 2.2% | 54.9% | 16.5% | 5.6% | 35.5% |  |
| Therapeutic massage | | 14.6% | 0.4% | 55.7% | 10.6% | 1.4% | 39.6% |  |
| No. of enrollees | | 142,225 | 2,454,766 |  | 98,512 | 98,512 |  |  |
| No. of enrollee-years | | 528,812 | 8,032,398 |  | 409,608 | 391,996 |  |  |

Abbreviations: SSBCI, special supplemental benefits for the chronically Ill; SD, standard deviation; AMI, acute myocardial infarction; COPD, chronic obstructive pulmonary disease; SNF, skilled nursing facility.

^a^ Other race is a category defined in the Master Beneficiary Summary File (MBSF).

^b^ Following the Chronic Condition Warehouse algorithm, two years of data are used to construct all conditions except for AMI, pneumonia, and stroke. ^c^ Arthritis includes rheumatoid arthritis or osteoarthritis.

^d^ Number suppressed because small sample size (N < 11) based on CMS privacy guidelines.

# Appendix Table A3.2. Descriptive statistics of study variables for the analyses of SSBCI: Dual eligible group

|  | Dual eligible group, Mean (SD) or % | | | | | | |  |
| --- | --- | --- | --- | --- | --- | --- | --- | --- |
|  | | Before matching | | | After matching | | | |
|  | | Benefit offering plans | Not-offering plans | Standardized difference | Benefit offering plans | Not-offering plans | Standardized difference | |
| Female | | 65.4% | 63.6% | 3.8% | 65.9% | 65.4% | 0.9% | |
| Age | |  |  |  |  |  |  | |
| Age < 65 | | 32.6% | 30.9% | 3.6% | 31.9% | 32.2% | -0.7% | |
| 65 ≤ Age < 70 | | 18.8% | 20.1% | -3.4% | 18.4% | 19.6% | -3.1% | |
| 70 ≤ Age < 75 | | 18.0% | 16.7% | 3.4% | 18.2% | 18.0% | 0.3% | |
| 75 ≤ Age < 80 | | 13.0% | 12.7% | 0.8% | 13.2% | 12.9% | 0.9% | |
| 80 ≤ Age < 85 | | 9.1% | 9.3% | -0.7% | 9.3% | 9.0% | 1.2% | |
| Age ≥ 85 | | 8.6% | 10.3% | -5.7% | 9.1% | 8.3% | 2.6% | |
| Race | |  |  |  |  |  |  | |
| Asian | | 7.8% | 5.9% | 7.3% | 7.9% | 7.7% | 0.8% | |
| Hispanic | | 21.8% | 22.0% | -0.5% | 22.5% | 23.4% | -2.2% | |
| Non-Hispanic Black | | 25.8% | 21.4% | 10.2% | 25.0% | 24.2% | 1.7% | |
| Non-Hispanic White | | 42.6% | 48.3% | -11.6% | 42.6% | 42.5% | 0.2% | |
| Other racea | | 2.1% | 2.3% | -1.2% | 2.1% | 2.2% | -0.9% | |
| Frailty score, Mean (SD) | | 0.19 (0.07) | 0.20 (0.07) | -8.3% | 0.20 (0.07) | 0.20 (0.07) | 2.3% | |
| Chronic Conditions^b^ | |  |  |  |  |  |  | |
| Anemia | | 26.5% | 28.9% | -5.4% | 26.8% | 26.5% | 0.5% | |
| Arthritis^c^ | | 47.1% | 45.7% | 2.7% | 47.6% | 47.0% | 1.2% | |
| Cancer | |  |  |  |  |  |  | |
| Breast | | 3.7% | 4.1% | -1.9% | 3.9% | 3.9% | 0.0% | |
| Colon | | 1.6% | 1.9% | -2.4% | 1.6% | 1.6% | -0.4% | |
| Endometrial | | 0.6% | 0.7% | -0.7% | 0.7% | 0.7% | 0.0% | |
| Lung | | 1.3% | 1.8% | -4.3% | 1.3% | 1.3% | 0.5% | |
| Prostate | | 2.5% | 2.7% | -1.5% | 2.5% | 2.5% | -0.4% | |
| Urologic | | 0.7% | 0.8% | -1.8% | 0.7% | 0.7% | -0.1% | |
| Cardiovascular disease | |  |  |  |  |  |  | |
| AMI | | 1.3% | 2.0% | -5.4% | 1.3% | 1.4% | -0.6% | |
| Atrial fibrillation | | 9.9% | 12.7% | -8.9% | 10.3% | 10.1% | 0.5% | |
| Ischemic heart disease | | 21.5% | 24.2% | -6.5% | 22.1% | 22.3% | -0.6% | |
| Chronic kidney disease | | 27.5% | 28.6% | -2.4% | 28.7% | 28.2% | 1.1% | |
| Dementia | |  |  |  |  |  |  | |
| Alzheimer’s | | 2.3% | 3.1% | -5.3% | 2.3% | 2.3% | 0.2% | |
| Non-Alzheimer’s | | 7.3% | 9.6% | -8.3% | 7.6% | 7.0% | 1.9% | |
| Depress | | 37.9% | 36.6% | 2.7% | 38.4% | 37.5% | 1.9% | |
| Diabetes | | 46.7% | 47.0% | -0.6% | 48.1% | 48.3% | -0.3% | |
| Heart failure | | 16.5% | 18.5% | -5.2% | 16.9% | 16.4% | 1.6% | |
| Lung diseases | |  |  |  |  |  |  | |
| Asthma | | 15.4% | 14.2% | 3.4% | 15.8% | 15.5% | 0.9% | |
| COPD | | 26.7% | 27.2% | -1.1% | 27.3% | 26.4% | 1.9% | |
| Pneumonia | | 5.4% | 7.9% | -10.0% | 5.5% | 5.9% | -1.4% | |
| Parkinson’s | | 1.5% | 1.7% | -1.7% | 1.5% | 1.5% | 0.5% | |
| Stroke | | 5.1% | 6.2% | -4.7% | 5.1% | 5.3% | -1.1% | |
| Enrollment in D-SNPs | |  |  |  |  |  |  | |
| Co-only D-SNPs | | 50.7% | 40.8% | 20.0% | 51.5% | 49.5% | 4.0% | |
| Integrated D-SNPs^d^ | | 27.6% | 13.8% | 34.6% | 26.1% | 23.0% | 7.2% | |
| County-level health care resources, Mean (SD) | | | | |  |  |  | |
| Beds/1000, No. | | 3.32 (2.19) | 3.04 (2.01) | 13.4% | 3.31 (2.17) | 3.21 (2.25) | 4.3% | |
| Doctors/1000, No. | | 3.93 (2.78) | 3.64 (2.65) | 10.7% | 3.96 (2.86) | 3.89 (2.89) | 2.5% | |
| SNF beds/1000, No. | | 5.17 (2.36) | 5.29 (2.83) | -4.7% | 5.18 (2.39) | 5.10 (2.70) | 3.3% | |
| Rural | | 14.0% | 18.1% | -11.1% | 14.0% | 14.8% | -2.1% | |
| ZIP code demographics, Mean (SD) | | | | |  |  |  | |
| College educated | | 26.25 (14.49) | 26.79 (14.75) | -3.7% | 26.24 (14.58) | 26.11 (14.25) | 0.9% | |
| Speaking English only | | 80.45 (24.21) | 80.93 (23.89) | -2.0% | 79.71 (24.26) | 78.78 (24.34) | 3.8% | |
| Under federal poverty level | | 17.64 (9.69) | 16.63 (9.04) | 10.8% | 17.64 (9.73) | 17.65 (9.48) | -0.2% | |
| Supplemental benefits | |  |  |  |  |  |  | |
| Medical transportation | | 80.1% | 62.4% | 40.0% | 78.8% | 77.4% | 3.4% | |
| Meals (a limited period) | | 68.5% | 58.2% | 21.6% | 65.6% | 63.5% | 4.4% | |
| Food security | | 6.6% | 0.3% | 35.1% | 7.0% | 1.1% | 30.0% | |
| Housing quality | | 3.8% | 0.8% | 19.9% | 1.8% | 1.9% | -1.1% | |
| Non-medical transportation | | 9.1% | 9.6% | -1.8% | 7.3% | 8.0% | -2.8% | |
| Social needs benefit | | 22.8% | 3.2% | 60.9% | 20.4% | 8.8% | 33.2% | |
| General supports for living | | 7.8% | 0.2% | 39.5% | 7.9% | 0.5% | 37.9% | |
| No. of enrollees | | 101,716 | 519,872 |  | 69,195 | 69,195 |  | |
| No. of enrollee-years | | 819,681 | 878,391 |  | 283,223 | 206,440 |  | |

Abbreviations: SSBCI, special supplemental benefits for the chronically Ill; SD, standard deviation; AMI, acute myocardial infarction; COPD, chronic obstructive pulmonary disease; SNF, skilled nursing facility; D-SNPs, dual-eligible special needs plans.

^a^ Other race is a category defined in the Master Beneficiary Summary File (MBSF).

^b^ Following the Chronic Condition Warehouse algorithm, two years of data are used to construct all conditions except for AMI, pneumonia, and stroke.

^c^ Arthritis includes rheumatoid arthritis and osteoarthritis.

^d^ Integrated D-SNPs include fully integrated dual-eligible special needs plans (FIDE) or highly integrated dual-eligible special needs plans (HIDE).

# Appendix Table A4.1. Descriptive statistics of study variables for the analyses of expanded PHR benefits among highly frail enrollees: Non-dual eligible group

|  | Non-dual eligible group, Mean (SD) or % | | | | | |
| --- | --- | --- | --- | --- | --- | --- |
|  | Before matching | | | After matching | | |
|  | Benefit offering plans | Not-offering plans | Standardized difference | Benefit offering plans | Not-offering plans | Standardized difference |
| Female | 61.0% | 58.0% | 5.4% | 61.0% | 61.0% | -0.3% |
| Age |  |  |  |  |  |  |
| Age < 65 | 8.0% | 6.0% | 8.8% | 9.0% | 8.0% | 1.2% |
| 65 ≤ Age < 70 | 13.0% | 13.0% | 0.4% | 14.0% | 14.0% | 0.8% |
| 70 ≤ Age < 75 | 21.0% | 21.0% | -0.9% | 21.0% | 21.0% | -1.6% |
| 75 ≤ Age < 80 | 22.0% | 21.0% | 0.5% | 21.0% | 22.0% | -1.0% |
| 80 ≤ Age < 85 | 18.0% | 18.0% | 0.2% | 17.0% | 17.0% | 0.0% |
| Age ≥ 85 | 19.0% | 21.0% | -5.8% | 18.0% | 18.0% | 1.2% |
| Race |  |  |  |  |  |  |
| Asian | 3.0% | 2.0% | 5.0% | 3.0% | 2.0% | 2.3% |
| Hispanic | 8.0% | 5.0% | 12.0% | 9.0% | 8.0% | 1.9% |
| Non-Hispanic Black | 7.0% | 10.0% | -10.8% | 7.0% | 8.0% | -2.3% |
| Non-Hispanic White | 80.0% | 81.0% | -2.0% | 79.0% | 80.0% | -0.6% |
| Other race^a^ | 2.0% | 2.0% | -0.1% | 2.0% | 2.0% | -0.3% |
| Frailty score, Mean (SD) | 0.20 (0.06) | 0.21 (0.07) | -11.2% | 0.20 (0.06) | 0.20 (0.06) | -2.6% |
| Chronic Conditions^b^ |  |  |  |  |  |  |
| Anemia | 27.0% | 30.0% | -8.0% | 26.0% | 27.0% | -1.8% |
| Arthritis^c^ | 48.0% | 49.0% | -2.2% | 47.0% | 48.0% | -1.7% |
| Cancer |  |  |  |  |  |  |
| Breast | 6.0% | 6.0% | -1.2% | 5.0% | 6.0% | -0.7% |
| Colon | 2.0% | 2.0% | -2.2% | 2.0% | 2.0% | 0.4% |
| Endometrial | 1.0% | 1.0% | -0.7% | 1.0% | 1.0% | 0.1% |
| Lung | 2.0% | 2.0% | -3.7% | 2.0% | 2.0% | 0.7% |
| Prostate | 5.0% | 6.0% | -3.6% | 5.0% | 5.0% | 0.3% |
| Urologic | 1.0% | 1.0% | -1.5% | 1.0% | 1.0% | -0.1% |
| Cardiovascular disease |  |  |  |  |  |  |
| AMI | 2.0% | 2.0% | -4.6% | 2.0% | 2.0% | -1.8% |
| Atrial fibrillation | 22.0% | 25.0% | -6.2% | 21.0% | 22.0% | -0.5% |
| Ischemic heart disease | 34.0% | 37.0% | -5.5% | 34.0% | 34.0% | -1.6% |
| Chronic kidney disease | 37.0% | 34.0% | 6.3% | 35.0% | 34.0% | 2.8% |
| Dementia |  |  |  |  |  |  |
| Alzheimer's | 3.0% | 5.0% | -6.6% | 3.0% | 3.0% | -0.4% |
| Non-Alzheimer's | 9.0% | 13.0% | -10.9% | 9.0% | 10.0% | -0.9% |
| Depress | 32.0% | 29.0% | 5.8% | 31.0% | 31.0% | 0.4% |
| Diabetes | 40.0% | 42.0% | -3.5% | 40.0% | 41.0% | -1.8% |
| Heart failure | 22.0% | 23.0% | -3.1% | 21.0% | 20.0% | 1.0% |
| Lung diseases |  |  |  |  |  |  |
| Asthma | 10.0% | 11.0% | -2.2% | 10.0% | 11.0% | -1.7% |
| COPD | 26.0% | 24.0% | 3.4% | 25.0% | 24.0% | 1.7% |
| Pneumonia | 5.0% | 8.0% | -9.2% | 5.0% | 6.0% | -3.7% |
| Parkinson's | 3.0% | 3.0% | -3.1% | 3.0% | 3.0% | 0.2% |
| Stroke | 6.0% | 7.0% | -4.3% | 6.0% | 7.0% | -1.2% |
| County-level health care resources, Mean (SD) | | | |  |  |  |
| Beds/1000, No. | 2.92 (1.84) | 2.94 (2.02) | -1.3% | 2.91 (1.76) | 2.90 (1.94) | 0.6% |
| Doctors/1000, No. | 3.52 (2.21) | 3.40 (2.29) | 5.6% | 3.54 (2.18) | 3.53 (2.35) | 0.2% |
| SNF beds/1000, No. | 5.15 (2.52) | 5.42 (2.76) | -10.2% | 5.22 (2.64) | 5.28 (2.66) | -2.3% |
| Rural | 12.0% | 18.0% | -15.7% | 12.0% | 13.0% | -1.9% |
| ZIP code demographics, Mean (SD) | | | |  |  |  |
| College educated | 31.04 (14.99) | 31.35 (16.12) | -2.0% | 31.02 (15.09) | 31.55 (15.93) | -3.4% |
| Speaking English only | 84.16 (19.10) | 86.16 (16.74) | -11.2% | 83.20 (19.78) | 84.00 (18.83) | -4.2% |
| Under federal poverty level | 12.19 (7.17) | 12.51 (7.64) | -4.3% | 12.27 (7.26) | 12.15 (7.32) | 1.7% |
| Supplemental benefits |  |  |  |  |  |  |
| Medical transportation | 43.0% | 13.0% | 71.4% | 39.0% | 24.0% | 33.8% |
| Meals (a limited period) | 45.0% | 24.0% | 45.8% | 39.0% | 31.0% | 16.5% |
| Food security | 9.0% | 1.0% | 37.3% | 9.0% | 1.0% | 34.4% |
| Housing quality | 3.0% | 0.0% | 25.0% | 3.0% | 0.0% | 20.8% |
| Non-medical transportation | 4.0% | 0.0% | 30.2% | 5.0% | 0.0% | 31.0% |
| Social needs benefit | 0.0% | 0.0% | 7.0% | 0.0% | 0.0% | 5.4% |
| General supports for living | 2.0% | 0.0% | 20.4% | 2.0% | 0.0% | 19.7% |
| No. of enrollees | 161,329 | 913,761 |  | 129,231 | 129,231 |  |
| No. of enrollee-years | 1,071,532 | 8,169,319 |  | 543,573 | 513,517 |  |

Abbreviations: PHR, primarily health-related; SD, standard deviation; AMI, acute myocardial infarction; COPD, chronic obstructive pulmonary disease; SNF, skilled nursing facility.

^a^ Other race is a category defined in the Master Beneficiary Summary File (MBSF).

^b^ Following the Chronic Condition Warehouse algorithm, two years of data are used to construct all conditions except for AMI, pneumonia, and stroke.

^c^ Arthritis includes rheumatoid arthritis and osteoarthritis.

# Appendix Table A4.2. Descriptive statistics of study variables for the analyses of expanded PHR benefits among highly frail enrollees: Dual eligible group

|  | Dual eligible group, Mean (SD) or % | | | | | |  |
| --- | --- | --- | --- | --- | --- | --- | --- |
|  | Before matching | | | After matching | | | |
|  | Benefit offering plans | Not-offering plans | Standardized difference | Benefit offering plans | Not-offering plans | Standardized difference | |
| Female | 71.0% | 69.0% | 4.4% | 71.0% | 71.0% | -0.4% | |
| Age |  |  |  |  |  |  | |
| Age < 65 | 31.0% | 26.0% | 9.4% | 32.0% | 32.0% | 0.2% | |
| 65 ≤ Age < 70 | 15.0% | 14.0% | 1.7% | 16.0% | 16.0% | -0.2% | |
| 70 ≤ Age < 75 | 17.0% | 16.0% | 2.6% | 16.0% | 16.0% | 0.0% | |
| 75 ≤ Age < 80 | 14.0% | 14.0% | 0.0% | 14.0% | 14.0% | -0.4% | |
| 80 ≤ Age < 85 | 11.0% | 12.0% | -4.4% | 11.0% | 11.0% | 0.3% | |
| Age ≥ 85 | 12.0% | 16.0% | -12.7% | 12.0% | 12.0% | -0.1% | |
| Race |  |  |  |  |  |  | |
| Asian | 4.0% | 6.0% | -8.1% | 3.0% | 4.0% | -1.4% | |
| Hispanic | 24.0% | 21.0% | 7.4% | 23.0% | 23.0% | 0.0% | |
| Non-Hispanic Black | 20.0% | 24.0% | -8.9% | 21.0% | 21.0% | 0.1% | |
| Non-Hispanic White | 51.0% | 48.0% | 5.7% | 51.0% | 51.0% | 1.0% | |
| Other race^a^ | 2.0% | 2.0% | -4.0% | 2.0% | 2.0% | -1.9% | |
| Frailty score, Mean (SD) | 0.24 (0.07) | 0.25 (0.08) | -9.8% | 0.24 (0.07) | 0.24 (0.07) | 2.1% | |
| Chronic Conditions^b^ |  |  |  |  |  |  | |
| Anemia | 35.0% | 40.0% | -10.3% | 36.0% | 36.0% | -0.2% | |
| Arthritis^c^ | 58.0% | 54.0% | 6.8% | 58.0% | 57.0% | 2.0% | |
| Cancer |  |  |  |  |  |  | |
| Breast | 4.0% | 5.0% | -1.9% | 4.0% | 4.0% | -0.2% | |
| Colon | 2.0% | 2.0% | -3.3% | 2.0% | 2.0% | 0.3% | |
| Endometrial | 1.0% | 1.0% | -1.2% | 1.0% | 1.0% | 0.2% | |
| Lung | 2.0% | 2.0% | -6.0% | 2.0% | 2.0% | 0.5% | |
| Prostate | 2.0% | 3.0% | -0.9% | 2.0% | 2.0% | 1.0% | |
| Urologic | 1.0% | 1.0% | -1.0% | 1.0% | 1.0% | 1.0% | |
| Cardiovascular disease |  |  |  |  |  |  | |
| AMI | 2.0% | 3.0% | -8.2% | 2.0% | 2.0% | -3.1% | |
| Atrial fibrillation | 16.0% | 20.0% | -9.1% | 16.0% | 16.0% | 0.3% | |
| Ischemic heart disease | 35.0% | 36.0% | -3.1% | 35.0% | 34.0% | 1.6% | |
| Chronic kidney disease | 40.0% | 38.0% | 3.8% | 39.0% | 36.0% | 5.8% | |
| Dementia |  |  |  |  |  |  | |
| Alzheimer's | 4.0% | 7.0% | -10.5% | 4.0% | 4.0% | -0.2% | |
| Non-Alzheimer's | 13.0% | 19.0% | -16.6% | 13.0% | 14.0% | -0.6% | |
| Depress | 52.0% | 45.0% | 15.2% | 52.0% | 50.0% | 4.7% | |
| Diabetes | 54.0% | 55.0% | -1.5% | 54.0% | 54.0% | 0.3% | |
| Heart failure | 28.0% | 31.0% | -6.6% | 28.0% | 27.0% | 1.1% | |
| Lung diseases |  |  |  |  |  |  | |
| Asthma | 19.0% | 20.0% | -2.4% | 19.0% | 20.0% | -1.8% | |
| COPD | 42.0% | 36.0% | 11.2% | 41.0% | 39.0% | 5.4% | |
| Pneumonia | 9.0% | 12.0% | -11.8% | 9.0% | 10.0% | -2.9% | |
| Parkinson's | 3.0% | 3.0% | -3.3% | 3.0% | 3.0% | 0.4% | |
| Stroke | 7.0% | 10.0% | -7.9% | 8.0% | 8.0% | -2.2% | |
| Enrollment in D-SNPs |  |  |  |  |  |  | |
| Co-only D-SNPs | 54.0% | 43.0% | 23.3% | 57.0% | 56.0% | 1.5% | |
| Integrated D-SNPs^d^ | 21.0% | 15.0% | 14.1% | 18.0% | 17.0% | 4.3% | |
| County-level health care resources, Mean (SD) | | | |  |  |  | |
| Beds/1000, No. | 3.13 (1.79) | 3.24 (2.10) | -5.9% | 3.16 (1.91) | 3.17 (2.20) | -0.7% | |
| Doctors/1000, No. | 3.56 (2.46) | 4.08 (2.99) | -18.9% | 3.53 (2.51) | 3.71 (2.69) | -6.6% | |
| SNF beds/1000, No. | 5.08 (2.44) | 5.24 (2.55) | -6.3% | 5.19 (2.56) | 5.29 (2.65) | -3.5% | |
| Rural | 15.0% | 14.0% | 4.0% | 17.0% | 16.0% | 0.4% | |
| ZIP code demographics, Mean (SD) | | | |  |  |  | |
| College educated | 25.52 (13.57) | 27.17 (15.01) | -11.5% | 25.31 (13.54) | 25.90 (14.18) | -4.3% | |
| Speaking English only | 77.68 (25.54) | 78.50 (23.45) | -3.3% | 78.15 (25.30) | 78.55 (24.13) | -1.6% | |
| Under federal poverty level | 16.85 (8.88) | 17.40 (9.71) | -5.9% | 17.12 (8.99) | 17.41 (9.48) | -3.1% | |
| Supplemental benefits |  |  |  |  |  |  | |
| Medical transportation | 81.0% | 57.0% | 53.0% | 80.0% | 75.0% | 13.0% | |
| Meals (a limited period) | 67.0% | 52.0% | 31.2% | 64.0% | 56.0% | 16.6% | |
| Food security | 13.0% | 8.0% | 16.5% | 14.0% | 7.0% | 22.1% | |
| Housing quality | 6.0% | 2.0% | 24.4% | 6.0% | 2.0% | 20.0% | |
| Non-medical transportation | 8.0% | 1.0% | 34.4% | 8.0% | 1.0% | 35.6% | |
| Social needs benefit | 1.0% | 0.0% | 4.0% | 1.0% | 0.0% | 7.8% | |
| General supports for living | 5.0% | 1.0% | 25.3% | 5.0% | 0.0% | 27.7% | |
| No. of enrollees | 46,752 | 150,656 |  | 35,983 | 35,983 |  | |
| No. of enrollee-years | 261,291 | 952,863 |  | 140,646 | 110,344 |  | |

Abbreviations: PHR, primarily health-related; SD, standard deviation; AMI, acute myocardial infarction; COPD, chronic obstructive pulmonary disease; SNF, skilled nursing facility; D-SNPS, dual-eligible special needs plans.

^a^ Other race is a category defined in the Master Beneficiary Summary File (MBSF).

^b^ Following the Chronic Condition Warehouse algorithm, two years of data are used to construct all conditions except for AMI, pneumonia, and stroke.

^c^ Arthritis includes rheumatoid arthritis and osteoarthritis.

^d^ Integrated D-SNPs include fully integrated dual-eligible special needs plans (FIDE) or highly integrated dual-eligible special needs plans (HIDE).

# Appendix Table A5.1. Descriptive statistics of study variables for the analyses of SSBCI among highly frail enrollees: Non-dual eligible group

|  | Non-dual eligible group, Mean (SD) or % | | | | | | |  |
| --- | --- | --- | --- | --- | --- | --- | --- | --- |
|  | | Before matching | | | After matching | | | |
|  | | Benefit offering plans | Not-offering plans | Standardized difference | Benefit offering plans | Not-offering plans | Standardized difference | |
| Female | | 61.1% | 58.9% | 4.5% | 60.0% | 60.6% | -1.2% | |
| Age | |  |  |  |  |  |  | |
| Age < 65 | | 8.3% | 6.5% | 6.6% | 9.1% | 9.9% | -3.0% | |
| 65 ≤ Age < 70 | | 11.1% | 12.3% | -3.6% | 12.2% | 13.3% | -3.5% | |
| 70 ≤ Age < 75 | | 18.4% | 19.8% | -3.6% | 18.8% | 19.4% | -1.5% | |
| 75 ≤ Age < 80 | | 20.8% | 21.4% | -1.4% | 20.4% | 20.3% | 0.4% | |
| 80 ≤ Age < 85 | | 18.7% | 18.1% | 1.8% | 17.9% | 17.2% | 2.0% | |
| Age ≥ 85 | | 22.7% | 22.0% | 1.7% | 21.7% | 19.9% | 4.3% | |
| Race | |  |  |  |  |  |  | |
| Asian | | 2.2% | 1.9% | 2.1% | 2.2% | 1.7% | 4.1% | |
| Hispanic | | 11.7% | 6.7% | 17.2% | 11.0% | 10.1% | 3.0% | |
| Non-Hispanic Black | | 13.4% | 9.0% | 14.0% | 11.4% | 11.5% | -0.2% | |
| Non-Hispanic White | | 71.2% | 80.6% | -22.1% | 73.9% | 75.3% | -3.2% | |
| Other race^a^ | | 1.5% | 1.8% | -1.9% | 1.4% | 1.5% | -0.4% | |
| Frailty score, Mean (SD) | | 0.22 (0.06) | 0.22 (0.07) | -4.8% | 0.22 (0.06) | 0.22 (0.06) | -1.1% | |
| Chronic Conditions^b^ | |  |  |  |  |  |  | |
| Anemia | | 33.8% | 33.3% | 1.2% | 33.3% | 32.2% | 2.2% | |
| Arthritis^c^ | | 51.0% | 51.9% | -1.7% | 51.5% | 52.1% | -1.3% | |
| Cancer | |  |  |  |  |  |  | |
| Breast | | 6.0% | 6.1% | -0.5% | 5.6% | 5.7% | -0.2% | |
| Colon | | 2.2% | 2.3% | -0.1% | 2.3% | 2.0% | 2.0% | |
| Endometrial | | 0.8% | 0.8% | 0.5% | 0.8% | 0.7% | 0.9% | |
| Lung | | 1.9% | 2.3% | -2.7% | 1.9% | 1.7% | 2.0% | |
| Prostate | | 5.6% | 5.7% | -0.4% | 5.4% | 5.1% | 1.2% | |
| Urologic | | 1.1% | 1.3% | -1.7% | 1.2% | 1.2% | 0.4% | |
| Cardiovascular disease | |  |  |  |  |  |  | |
| AMI | | 1.9% | 2.5% | -3.7% | 2.0% | 2.2% | -1.0% | |
| Atrial fibrillation | | 24.6% | 26.4% | -4.2% | 24.8% | 23.8% | 2.1% | |
| Ischemic heart disease | | 40.1% | 39.7% | 0.7% | 40.1% | 39.9% | 0.5% | |
| Chronic kidney disease | | 42.4% | 38.1% | 8.8% | 40.2% | 38.8% | 2.8% | |
| Dementia | |  |  |  |  |  |  | |
| Alzheimer's | | 5.1% | 5.7% | -2.5% | 5.0% | 4.9% | 0.4% | |
| Non-Alzheimer's | | 13.5% | 15.3% | -5.0% | 13.1% | 12.8% | 1.1% | |
| Depress | | 34.3% | 33.8% | 1.0% | 34.0% | 35.0% | -2.0% | |
| Diabetes | | 46.8% | 44.6% | 4.5% | 46.2% | 46.7% | -1.1% | |
| Heart failure | | 28.2% | 25.9% | 5.3% | 26.8% | 26.1% | 1.7% | |
| Lung diseases | |  |  |  |  |  |  | |
| Asthma | | 10.9% | 11.9% | -3.1% | 11.0% | 11.5% | -1.8% | |
| COPD | | 28.5% | 27.9% | 1.5% | 28.5% | 28.6% | -0.2% | |
| Pneumonia | | 6.5% | 8.7% | -8.2% | 7.0% | 7.5% | -2.1% | |
| Parkinson's | | 3.2% | 3.7% | -2.8% | 3.1% | 3.2% | -0.6% | |
| Stroke | | 8.4% | 8.4% | -0.1% | 8.4% | 8.0% | 1.1% | |
| County-level health care resources, Mean (SD) | | | | |  |  |  | |
| Beds/1000, No. | | 3.04 (1.80) | 2.90 (1.90) | 7.5% | 3.08 (2.21) | 3.05 (2.08) | 1.3% | |
| Doctors/1000, No. | | 3.90 (2.30) | 3.41 (2.21) | 21.7% | 3.79 (2.66) | 3.64 (2.40) | 6.0% | |
| SNF beds/1000, No. | | 4.92 (2.28) | 5.32 (2.75) | -15.8% | 5.39 (2.39) | 5.47 (3.13) | -2.9% | |
| Rural | | 8.4% | 16.3% | -24.0% | 12.0% | 12.9% | -2.7% | |
| ZIP code demographics, Mean (SD) | | | | |  |  |  | |
| College educated | | 31.73 (15.68) | 31.38 (15.92) | 2.2% | 30.73 (15.62) | 30.34 (15.53) | 2.5% | |
| Speaking English only | | 79.56 (22.91) | 85.49 (17.79) | -28.9% | 81.92 (21.92) | 83.95 (19.30) | -9.8% | |
| Under federal poverty level | | 12.90 (8.07) | 12.38 (7.45) | 6.7% | 12.96 (8.26) | 13.04 (7.76) | -1.0% | |
| Supplemental benefits | |  |  |  |  |  |  | |
| Medical transportation | | 48.9% | 18.3% | 68.3% | 49.2% | 39.0% | 20.7% | |
| Meals (a limited period) | | 52.7% | 26.9% | 54.7% | 51.0% | 48.0% | 5.9% | |
| Adult day health | | 7.3% | -^d^ | - | 9.1% | - | - | |
| Home-based palliative care | | 11.3% | 1.6% | 40.2% | 9.0% | 7.0% | 7.5% | |
| Support for caregivers | | 5.1% | 4.7% | 1.5% | 4.3% | 2.2% | 12.1% | |
| In-home support | | 15.5% | 1.9% | 49.5% | 15.9% | 6.2% | 31.1% | |
| Therapeutic massage | | 13.1% | 0.3% | 52.7% | 10.5% | 1.3% | 39.7% | |
| No. of enrollees | | 40,578 | 1,097,062 |  | 28,850 | 28,850 |  | |
| No. of enrollee-year | | 262,093 | 8,566,979 |  | 127,775 | 118,603 |  | |

Abbreviations: SSBCI, special supplemental benefits for the chronically Ill; SD, standard deviation; AMI, acute myocardial infarction; COPD, chronic obstructive pulmonary disease; SNF, skilled nursing facility.

^a^ Other race is a category defined in the Master Beneficiary Summary File (MBSF).

^b^ Following the Chronic Condition Warehouse algorithm, two years of data are used to construct all conditions except for AMI, pneumonia, and stroke.

^c^ Arthritis includes rheumatoid arthritis and osteoarthritis.

^d^ Number suppressed because small sample size (N < 11) based on CMS privacy guidelines.

# Appendix Table A5.2. Descriptive statistics of study variables for the analyses of SSBCI among highly frail enrollees: Dual eligible group

|  | Dual eligible group, Mean (SD) or % | | | | | |  |
| --- | --- | --- | --- | --- | --- | --- | --- |
|  | Before matching | | | After matching | | | |
|  | Benefit offering plans | Not-offering plans | Standardized difference | Benefit offering plans | Not-offering plans | Standardized difference | |
| Female | 72.5% | 69.7% | 6.2% | 71.9% | 71.5% | 1.0% | |
| Age |  |  |  |  |  |  | |
| Age < 65 | 30.4% | 28.0% | 5.3% | 29.2% | 30.4% | -2.7% | |
| 65 ≤ Age < 70 | 15.7% | 15.6% | 0.4% | 15.9% | 17.0% | -3.1% | |
| 70 ≤ Age < 75 | 16.9% | 15.7% | 3.3% | 16.7% | 16.4% | 0.9% | |
| 75 ≤ Age < 80 | 13.9% | 14.1% | -0.7% | 14.1% | 13.8% | 0.8% | |
| 80 ≤ Age < 85 | 10.9% | 11.7% | -2.7% | 11.2% | 10.4% | 2.6% | |
| Age ≥ 85 | 12.2% | 14.9% | -7.9% | 12.9% | 11.9% | 2.9% | |
| Race |  |  |  |  |  |  | |
| Asian | 4.3% | 3.7% | 3.0% | 4.4% | 4.5% | -0.5% | |
| Hispanic | 20.2% | 20.8% | -1.6% | 21.6% | 21.4% | 0.3% | |
| Non-Hispanic Black | 28.2% | 21.5% | 15.4% | 24.9% | 24.0% | 2.2% | |
| Non-Hispanic White | 45.7% | 52.1% | -12.9% | 47.4% | 48.4% | -2.0% | |
| Other race^a^ | 1.7% | 1.8% | -1.1% | 1.7% | 1.7% | 0.1% | |
| Frailty score, Mean (SD) | 0.25 (0.07) | 0.26 (0.08) | -6.4% | 0.25 (0.07) | 0.25 (0.07) | -1.5% | |
| Chronic Conditions^b^ |  |  |  |  |  |  | |
| Anemia | 39.5% | 41.3% | -3.6% | 39.3% | 39.3% | -0.1% | |
| Arthritis^c^ | 60.4% | 57.6% | 5.6% | 60.1% | 60.0% | 0.1% | |
| Cancer |  |  |  |  |  |  | |
| Breast | 4.6% | 4.9% | -1.4% | 4.5% | 4.4% | 0.1% | |
| Colon | 1.8% | 2.2% | -3.1% | 1.8% | 1.9% | -0.8% | |
| Endometrial | 0.9% | 0.9% | -0.8% | 0.9% | 0.9% | 0.1% | |
| Lung | 1.9% | 2.5% | -3.8% | 2.0% | 1.8% | 0.9% | |
| Prostate | 2.3% | 2.5% | -1.2% | 2.3% | 2.3% | -0.4% | |
| Urologic | 1.0% | 1.1% | -1.1% | 0.9% | 1.0% | -0.9% | |
| Cardiovascular disease |  |  |  |  |  |  | |
| AMI | 2.5% | 3.4% | -5.7% | 2.6% | 2.7% | -0.6% | |
| Atrial fibrillation | 17.1% | 20.3% | -8.1% | 17.4% | 17.3% | 0.2% | |
| Ischemic heart disease | 37.0% | 38.8% | -3.8% | 36.8% | 37.3% | -1.0% | |
| Chronic kidney disease | 39.5% | 41.1% | -3.2% | 40.0% | 39.3% | 1.5% | |
| Dementia |  |  |  |  |  |  | |
| Alzheimer's | 5.5% | 6.5% | -4.5% | 5.7% | 5.6% | 0.5% | |
| Non-Alzheimer's | 16.3% | 18.9% | -6.8% | 17.0% | 16.4% | 1.7% | |
| Depress | 52.8% | 49.8% | 6.1% | 53.0% | 52.7% | 0.5% | |
| Diabetes | 57.6% | 56.5% | 2.2% | 57.5% | 57.2% | 0.6% | |
| Heart failure | 31.1% | 32.7% | -3.4% | 30.7% | 30.9% | -0.4% | |
| Lung diseases |  |  |  |  |  |  | |
| Asthma | 22.3% | 19.5% | 6.8% | 21.5% | 21.2% | 0.7% | |
| COPD | 41.7% | 41.4% | 0.7% | 41.0% | 41.1% | -0.1% | |
| Pneumonia | 10.3% | 13.5% | -9.9% | 10.7% | 11.5% | -2.5% | |
| Parkinson's | 3.2% | 3.3% | -0.7% | 3.3% | 3.1% | 1.1% | |
| Stroke | 8.9% | 9.9% | -3.5% | 8.9% | 9.6% | -2.2% | |
| Enrollment in D-SNPs |  |  |  |  |  |  | |
| Co-only D-SNPs | 55.3% | 45.0% | 20.7% | 52.6% | 54.7% | -4.3% | |
| Integrated D-SNPs^d^ | 29.5% | 13.2% | 40.8% | 29.5% | 24.8% | 10.5% | |
| County-level health care resources, Mean (SD) | | | |  |  |  | |
| Beds/1000, No. | 3.41 (2.23) | 3.07 (2.01) | 16.3% | 3.31 (2.10) | 3.24 (2.12) | 3.2% | |
| Doctors/1000, No. | 3.99 (2.80) | 3.56 (2.55) | 16.3% | 3.93 (2.93) | 3.84 (2.88) | 3.1% | |
| SNF beds/1000, No. | 5.14 (2.28) | 5.29 (2.80) | -5.7% | 5.29 (2.41) | 5.19 (2.81) | 3.7% | |
| Rural | 13.5% | 18.2% | -13.0% | 14.6% | 16.1% | -4.0% | |
| ZIP code demographics, Mean (SD) | | | |  |  |  | |
| College educated | 26.04 (14.30) | 26.15 (14.35) | -0.8% | 26.19 (14.38) | 25.95 (14.01) | 1.7% | |
| Speaking English only | 81.20 (22.97) | 80.10 (23.99) | 4.7% | 80.70 (23.59) | 79.81 (23.78) | 3.8% | |
| Under federal poverty level | 18.03 (9.85) | 16.80 (9.03) | 13.0% | 17.66 (9.73) | 17.72 (9.61) | -0.6% | |
| Supplemental benefits |  |  |  |  |  |  | |
| Medical transportation | 78.1% | 64.1% | 31.4% | 76.9% | 79.1% | -5.2% | |
| Meals (a limited period) | 65.0% | 57.7% | 14.9% | 63.4% | 64.2% | -1.7% | |
| Food security | 5.4% | 0.4% | 30.3% | 7.1% | 1.5% | 27.9% | |
| Housing quality | 3.9% | 0.8% | 20.4% | 1.8% | 1.8% | -0.2% | |
| Non-medical transportation | 8.7% | 11.8% | -10.2% | 7.7% | 9.3% | -5.8% | |
| Social needs benefit | 20.8% | 3.0% | 57.4% | 19.6% | 9.0% | 30.8% | |
| General supports for living | 6.5% | 0.2% | 35.6% | 7.9% | 0.5% | 37.5% | |
| No. of enrollees | 31,167 | 233,573 |  | 20,915 | 20,915 |  | |
| No. of enrollee-year | 197,722 | 1,291,666 |  | 91,320 | 63,989 |  | |

Abbreviations: SSBCI, special supplemental benefits for the chronically Ill; SD, standard deviation; AMI, acute myocardial infarction; COPD, chronic obstructive pulmonary disease; SNF, skilled nursing facility; D-SNPs, dual-eligible special needs plans.

^a^ Other race is a category defined in the Master Beneficiary Summary File (MBSF).

^b^ Following the Chronic Condition Warehouse algorithm, two years of data are used to construct all conditions except for AMI, pneumonia, and stroke.

^c^ Arthritis includes rheumatoid arthritis and osteoarthritis.

^d^ Integrated D-SNPs include fully integrated dual-eligible special needs plans (FIDE) or highly integrated dual-eligible special needs plans (HIDE).

# Appendix Table A6.1. Difference-in-differences estimates from benefit-specific analyses

|  | Non-dual eligible enrollees | | | Dual eligible enrollees | | |
| --- | --- | --- | --- | --- | --- | --- |
|  | N | Baseline mean (%) | Estimate (95% CI), % point | N | Baseline mean (%) | Estimate (95% CI), % point |
| *Offering in-home support services^a^* | | | | | | |
| Prob. of ED use^b^ | 983,157 | 29.1 | **-0.84 (-1.30, -0.37)^***^** | 340,407 | 42.9 | -0.54 (-1.13, 0.06) |
| Prob. of hospitalization^b^ | 983,157 | 13.7 | **-0.28 (-0.56, 0.00)^*^** | 340,407 | 18.4 | **-0.58 (-1.07, -0.09)^*^** |
| Prob. of re-admission^b^ | 45,717 | 14.4 | -0.43 (-2.33, 1.47) | 24,134 | 17.7 | 1.14 (-1.53, 3.81) |
| *Offering the food security benefit^c^* | | | | | | |
| Prob. of ED use | 378,319 | 32.0 | -1.33 (-2.07, -0.59)^***^ | 261,790 | 47.0 | -0.35 (-1.09, 0.40) |
| Prob. of hospitalization | 378,319 | 17.3 | -1.40 (-2.07, -0.73)^***^ | 261,790 | 23.4 | -0.13 (-0.75, 0.48) |
| Prob. of re-admission | 23,532 | 15.6 | -3.19 (-6.39, 0.01) | 25,891 | 20.3 | 1.92 (-0.40, 4.24) |
| *Offering the housing quality benefit^d^* | | | | | | |
| Prob. of ED use | 245,662 | 29.5 | -0.11 (-1.56, 1.35) | 228,699 | 44.1 | 0.80 (-0.15, 1.76) |
| Prob. of hospitalization | 245,662 | 14.8 | -0.35 (-1.20, 0.51) | 228,699 | 19.3 | 0.30 (-0.48, 1.08) |
| Prob. of re-admission | 14,792 | 15.8 | 2.28 (-5.83, 10.38) | 16,947 | 19.7 | 2.36 (-1.78, 6.51) |

Abbreviations: CI, confidence interval; %points, percentage points; ED, emergency department.

^a^ The sample for in-home support services analysis comprises enrollees with a chronic condition(s).

^b^ Annual probability of having an ED visit, hospitalization, or re-admission.

^c^ Food security benefits provide food/produce and meals not limited to post-discharges. The sample for this analysis comprises enrollees with a diet-sensitive condition (diabetes, cardiovascular disease, or chronic heart failure).

^d^ Housing quality benefits include pest control, indoor air quality, and structural modification. The sample for this analysis comprises enrollees with a chronic condition(s).

***, P-value < 0.001, **, P-value < 0.01, and *, P-value < 0.05.

Notes: Estimates of the overall treatment effects obtained from staggered difference-in-differences models using the matched sample, controlling for plan-county fixed effects, year dummies, enrollee demographics and health risks, area socio-economic factors and health resource variables, and time-varying plan attributes, including supplemental benefits other than the benefit in question. Standard errors are clustered at the plan-county level. **Bolded results are statistically significant and supported by parallel trends during the pre-treatment period.**

# Appendix Table A6.2. Difference-in-differences estimates from benefit-specific analyses among highly frail enrollees

|  | Non-dual eligible enrollees | | | Dual eligible enrollees | | |
| --- | --- | --- | --- | --- | --- | --- |
|  | N | Baseline mean (%) | Estimate (95% CI), % point | N | Baseline mean (%) | Estimate (95% CI), % point |
| *Offering in-home support services^a^* | | | | | | |
| Prob. of ED use^b^ | 315,997 | 56.3 | -4.80 (-5.59, -4.02)^***^ | 99,384 | 70.7 | -4.83 (-5.93, -3.72)^***^ |
| Prob. of hospitalization^b^ | 315,997 | 32.9 | **-1.89 (-2.58, -1.19)^***^** | 99,384 | 42.2 | **-2.36 (-3.59, -1.13)^***^** |
| Prob. of re-admission^b^ | 14,158 | 26.9 | -0.65 (-4.28, 2.98) | 7,154 | 33.5 | 0.29 (-5.05, 5.64) |
| *Offering the food security benefit^c^* | | | | | | |
| Prob. of ED use | 106,100 | 61.7 | -5.10 (-6.67, -3.53)^***^ | 80,360 | 75.6 | **-2.38 (-3.73, -1.02)^**^** |
| Prob. of hospitalization | 106,100 | 41.9 | **-5.05 (-6.42, -3.68)^***^** | 80,360 | 50.1 | -0.94 (-2.31, 0.42) |
| Prob. of re-admission | 6,511 | 30.4 | **-6.47 (-12.78, -0.17)^*^** | 8,237 | 34.3 | 2.43 (-2.50, 7.36) |
| *Offering the housing quality benefit^d^* | | | | | | |
| Prob. of ED use | 99,789 | 55.6 | **-3.53 (-6.65, -0.41)^*^** | 68,172 | 72.7 | **-2.31 (-4.15, -0.47)^*^** |
| Prob. of hospitalization | 99,789 | 33.6 | **-2.72 (-5.38, -0.06)^*^** | 68,172 | 44.8 | -1.13 (-2.94, 0.68) |
| Prob. of re-admission | 3,874 | 30.2 | **19.14 (4.14, 34.14)^*^** | 3,881 | 29.2 | 13.94 (-1.73, 29.60) |

Abbreviations: CI, confidence interval; %points, percentage points; ED, emergency department.

^a^ The sample for in-home support services analysis comprises enrollees with a chronic condition(s).

^b^ Annual probability of having an ED visit, hospitalization, or re-admission.

^c^ Food security benefits: food/produce and meals not limited to post-discharges. The sample for this analysis comprises enrollees with a diet-sensitive condition (diabetes, cardiovascular disease, or chronic heart failure).

^d^ Housing quality benefits: pest control, indoor air quality, or structural modification. The sample for this analysis includes enrollees with a chronic condition(s).

***, P-value < 0.001, **, P-value < 0.01, and *, P-value < 0.05.

Notes: Estimates of the overall treatment effects obtained from staggered difference-in-differences models using the matched sample, controlling for plan-county fixed effects, year dummies, enrollee demographics and health risks, area socio-economic factors and health resource variables, and time-varying plan attributes, including supplemental benefits other than the benefit in question. Standard errors are clustered at the plan-county level. **Bolded results are statistically significant and supported by parallel trends during the pre-treatment period.**

# Appendix Figure A1.1 Growth in the adoption of expanded supplemental benefits over the study period (2017-2022)


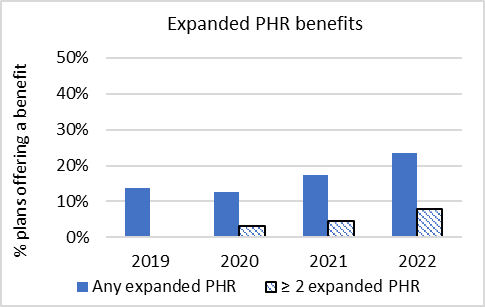

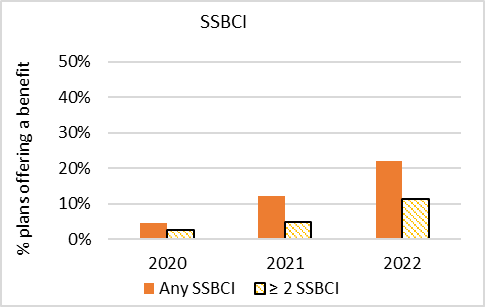


Abbreviations: PHR, primarily health-related; SSBCI, special supplemental benefits for the chronically Ill.

# Appendix Figure A1.2 Growth in the adoption of a single benefit or benefit group over the study period (2017-2022)


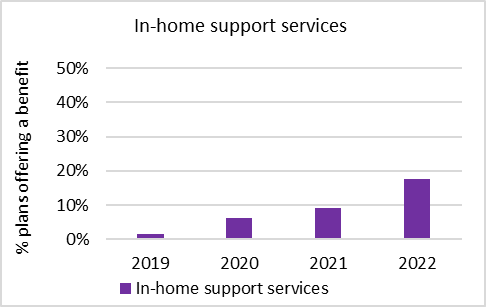

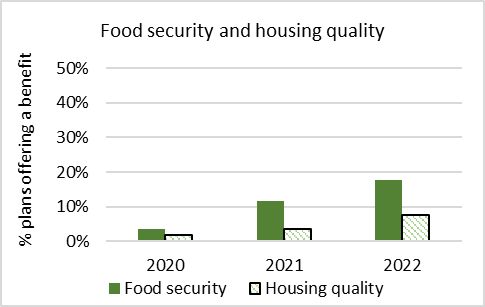


Note: In-home support services is an expanded primary health-related (PHR) supplemental benefit.

Food security benefits, including food/produce or meals not limited to post-discharges, are Special Supplemental Benefits for the Chronically Ill (SSBCI).

Housing quality benefits, including pest control, indoor air quality, or structural modification, are Special Supplemental Benefits for the Chronically Ill (SSBCI).

# Appendix Figure A2. Event-study plots from the analysis of offering any SSBCI

| **Panel A. Non-dual eligible enrollees** |
| --- |
| 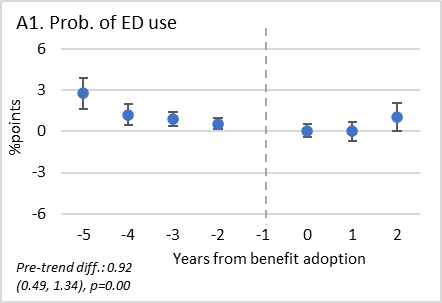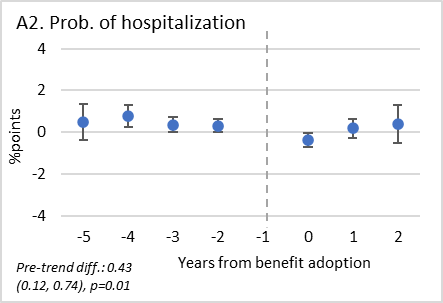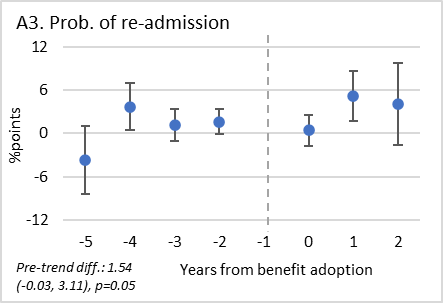 |
| **Panel B. Dual eligible enrollees** |
| 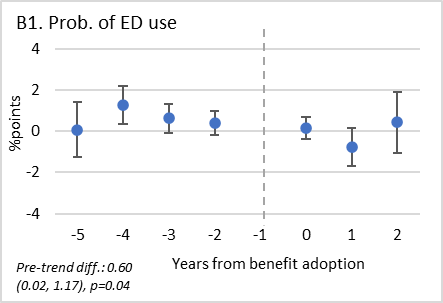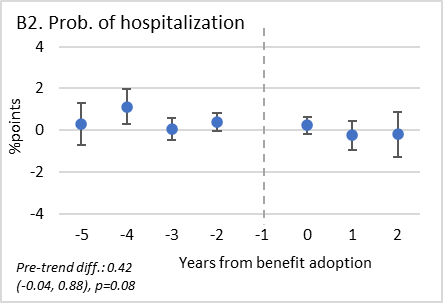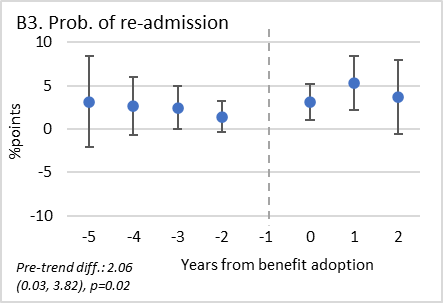 |

Abbreviations: SSBCI, special supplemental benefits for the chronically Ill; ED, emergency department; %points, percentage points; pre-trend diff., pre-treatment trends difference.

Note: Pre-trend diff. reports the average differential change in the outcome between benefit-offering plans and non-offering plans during the pre-treatment period. The outcome was the annual probability of having an adverse health event (ED visit, hospitalization, or re-admission). The unit of Y-axis is one percentage point. 95% confidence intervals are in parentheses, and the p-value corresponds to the parallel trends test during the pre-treatment period. In graphs, error bars indicate the 95% CIs. Year −1 is the omitted reference period.

Appendix Figure A3. Event-study plots from the analysis of offering ≥2 SSBCI

| **Panel A. Non-dual eligible enrollees** |
| --- |
| 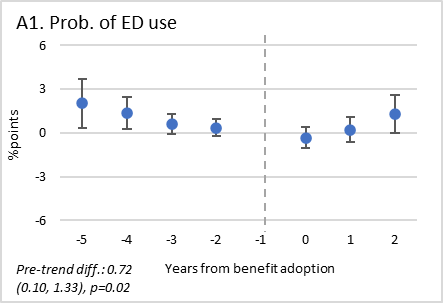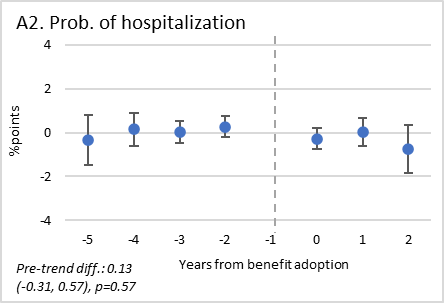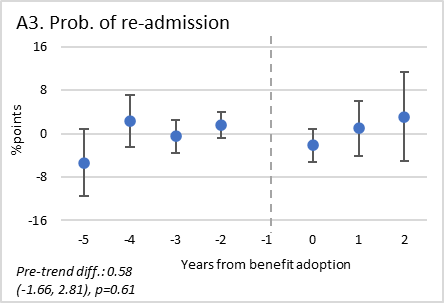 |
| **Panel B. Dual eligible enrollees** |
| 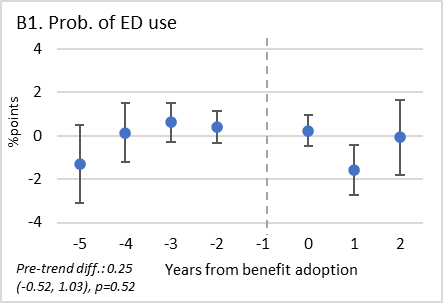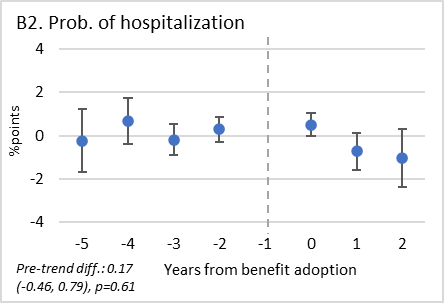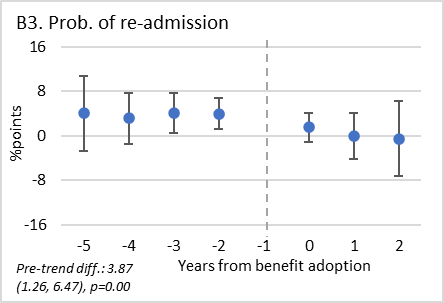 |

Abbreviations: SSBCI, special supplemental benefits for the chronically Ill; ED, emergency department; %points, percentage points; pre-trend diff., pre-treatment trends difference.

Note: Pre-trend diff. reports the average differential change in the outcome between benefit-offering plans and non-offering plans during the pre-treatment period. The outcome was the annual probability of having an adverse health event (ED visit, hospitalization, or re-admission). The unit of Y-axis is one percentage point. 95% confidence intervals are in parentheses, and the p-value corresponds to the parallel trends test during the pre-treatment period. In graphs, error bars indicate the 95% CIs. Year −1 is the omitted reference period.

Appendix Figure A4. *HonestDiD* results for the effect of offering any expanded PHR benefit on the annual probability of a re-admission for non-dual enrollees


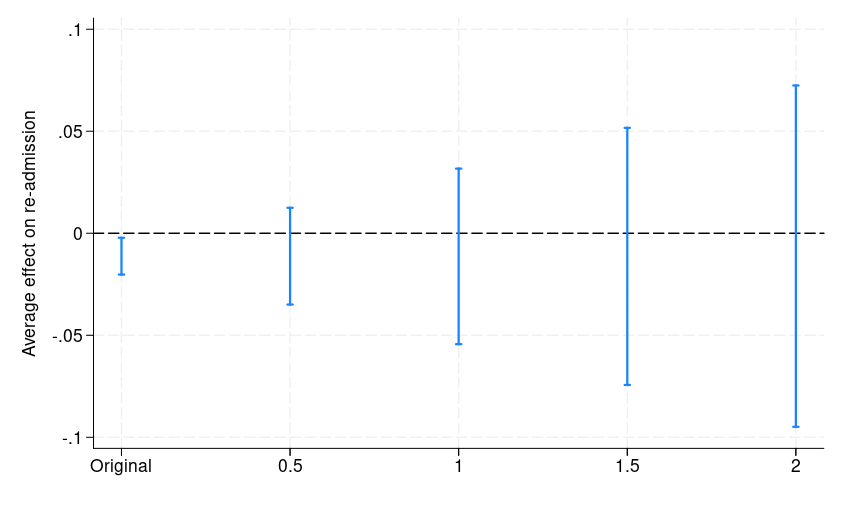


Abbreviations: PHR, primarily health-related.

Notes: Y-axis in the figure represents the annual probability of a re-admission. The figure presents the results from the HonestDiD method, which allows bounded deviations from parallel trends. Specifically, the method constrains post-treatment deviations to be bounded in magnitude relative to the deviations observed in the pre-treatment period and constructs confidence intervals that remain valid under these restrictions. The relative magnitude (M) to the pre-trend deviation is represented in the x-axis. For example, a value of 0.5 means that the post-treatment deviation from parallel trends is no greater than 50% of the pre-treatment period deviation; and a value of 1 means that the post-treatment deviation is no more than the pre-trend deviation. The figure shows the average treatment effect under different assumptions about the relative magnitude bounds to deviations from parallel trends (M = 0.5, 1, 1.5, 2 times the maximum pre-trend deviation).

Appendix Figure A5. *HonestDiD* results for the effect of offering any SSBCI on the annual probability of a re-admission for dual-eligible enrollees


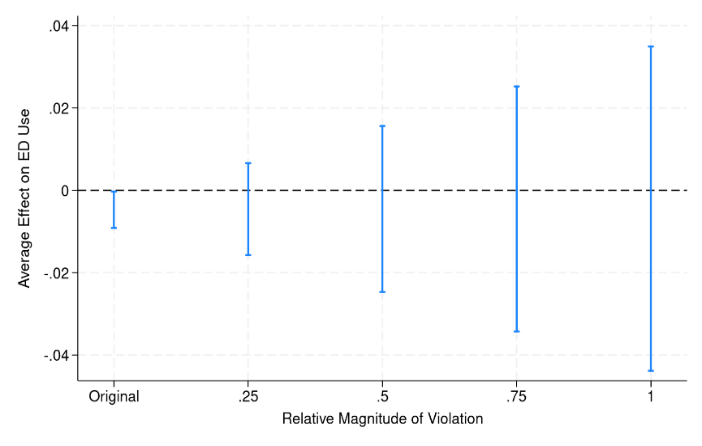

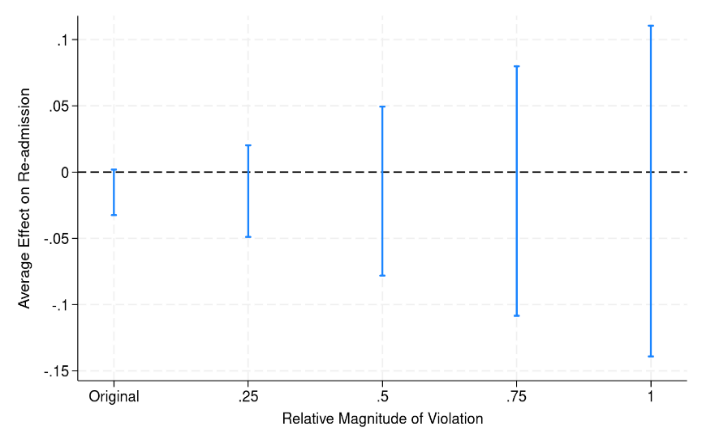

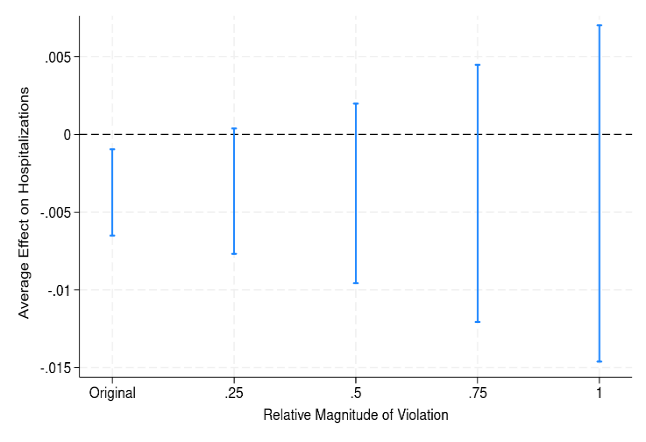

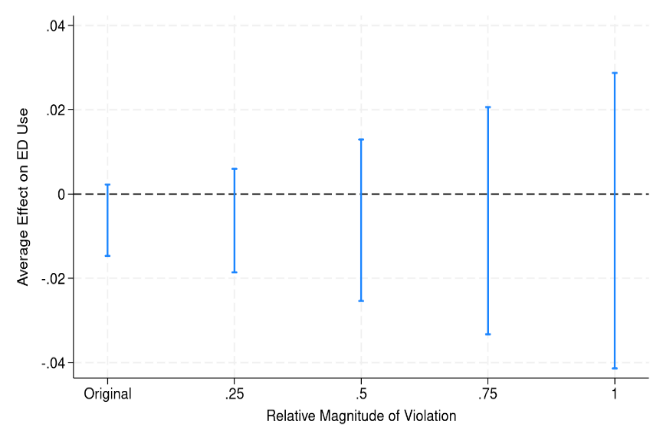

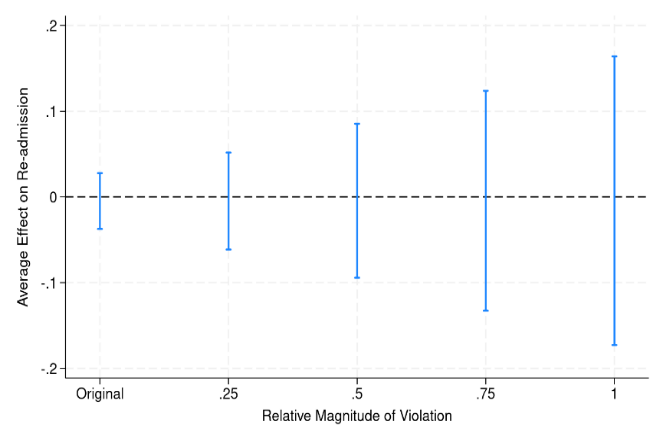

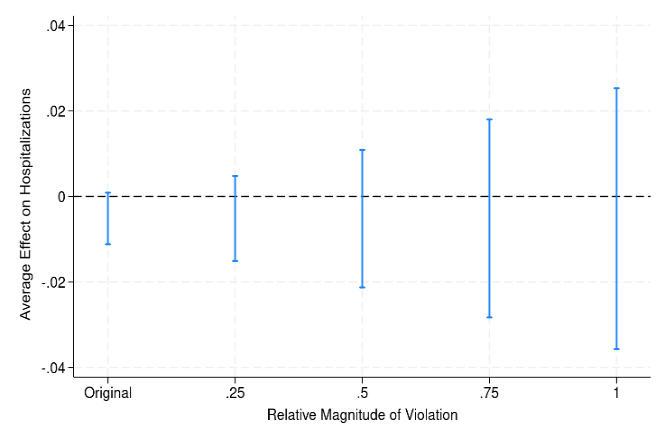


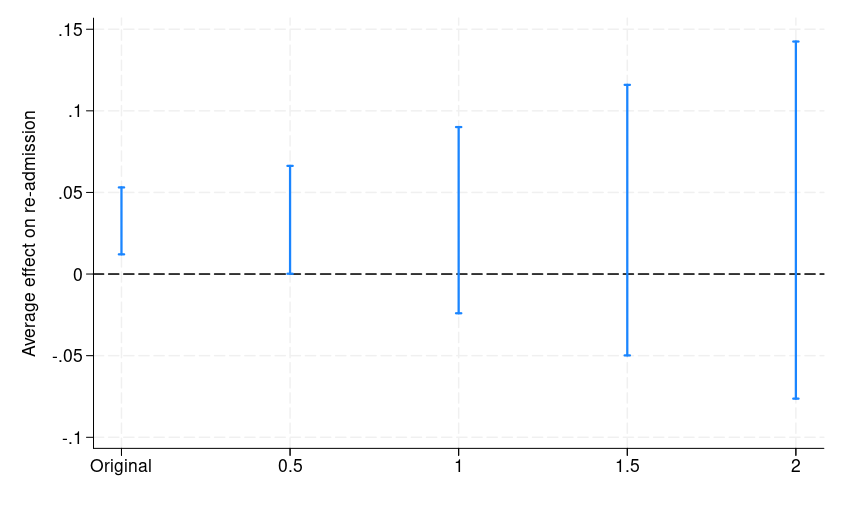


Abbreviations: SSBCI, special supplemental benefits for the chronically Ill.

Notes: Y-axis in the figure represents the annual probability of a re-admission. The figure presents the results from the HonestDiD method, which allows bounded deviations from parallel trends. Specifically, the method constrains post-treatment deviations to be bounded in magnitude relative to the deviations observed in the pre-treatment period and constructs confidence intervals that remain valid under these restrictions. The relative magnitude (M) to the pre-trend deviation is represented in the x-axis. For example, a value of 0.5 means that the post-treatment deviation from parallel trends is no greater than 50% of the pre-treatment period deviation; and a value of 1 means that the post-treatment deviation is no more than the pre-trend deviation. The figure shows the average treatment effect under different assumptions about the relative magnitude bounds to deviations from parallel trends (M = 0.5, 1, 1.5, 2 times the maximum pre-trend deviation).

Appendix Figure A6. Event-study plots from the analysis of any expanded PHR benefit among highly frail enrollees

| **Panel A. Non-dual eligible enrollees** |
| --- |
| 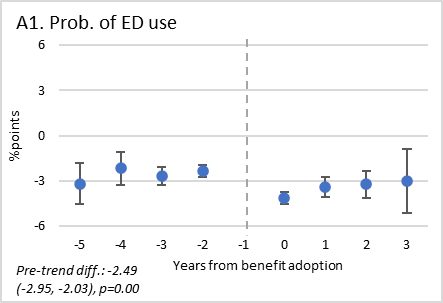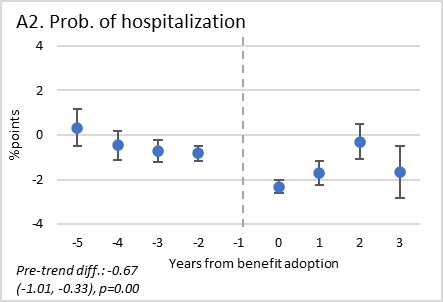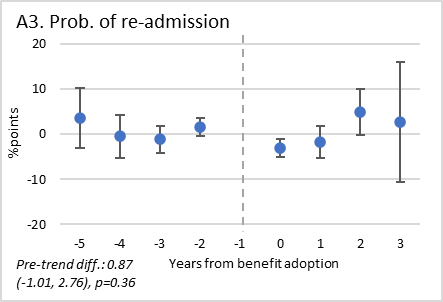 |
| **Panel B. Dual eligible enrollees** |
| 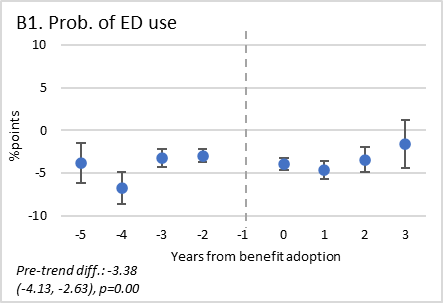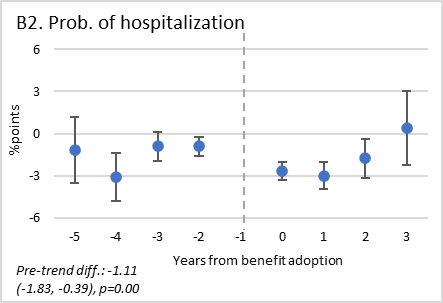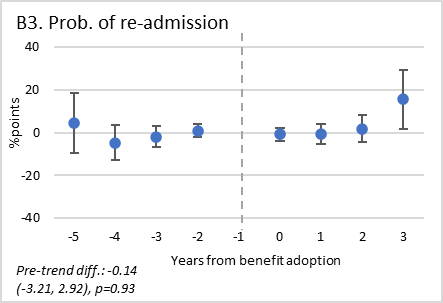 |

Abbreviations: PHR, primarily health-related; ED, emergency department; %points, percentage points; pre-trend diff., pre-treatment trends difference.

Notes: Pre-trend diff. reports the average differential change in the outcome between benefit-offering plans and non-offering plans during the pre-treatment period. The outcome was the annual probability of having an adverse health event (ED visit, hospitalization, or re-admission). The unit of Y-axis is one percentage point. 95% confidence intervals are in parentheses, and the p-value corresponds to the parallel trends test during the pre-treatment period. In graphs, error bars indicate the 95% CIs. Year −1 is the omitted reference period.

Appendix Figure A7. Event-study plots from the analysis of ≥ 2 expanded PHR benefits among highly frail enrollees

| **Panel A. Non-dual eligible enrollees** |
| --- |
| 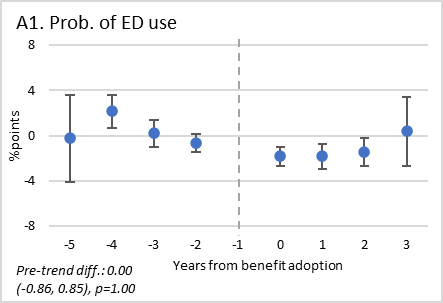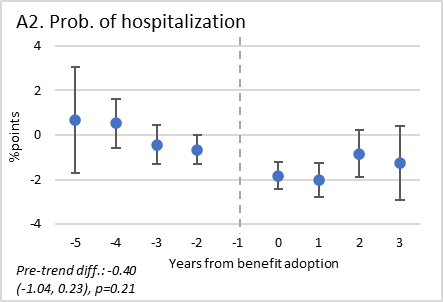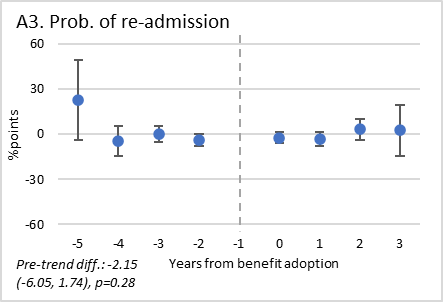 |
| **Panel B. Dual eligible enrollees** |
| 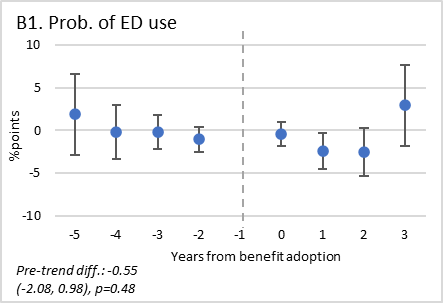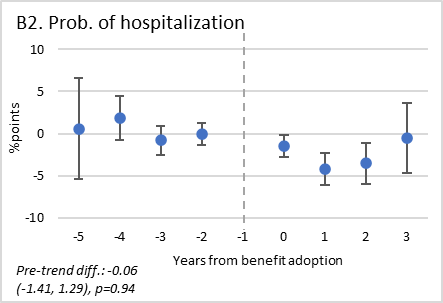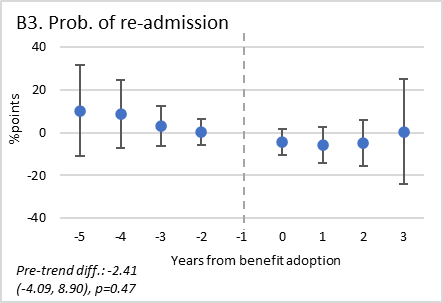 |

Abbreviations: PHR, primarily health-related; ED, emergency department; %points, percentage points; pre-trend diff., pre-treatment trends difference.

Notes: Pre-trend diff. reports the average differential change in the outcome between benefit-offering plans and non-offering plans during the pre-treatment period. The outcome was the annual probability of having an adverse health event (ED visit, hospitalization, or re-admission). The unit of Y-axis is one percentage point. 95% confidence intervals are in parentheses, and the p-value corresponds to the parallel trends test during the pre-treatment period. In graphs, error bars indicate the 95% CIs. Year −1 is the omitted reference period.


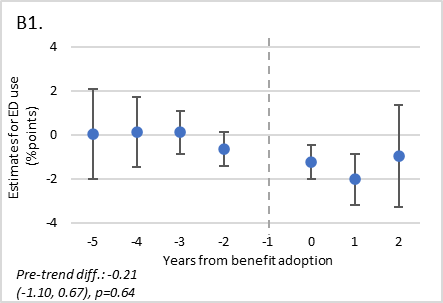

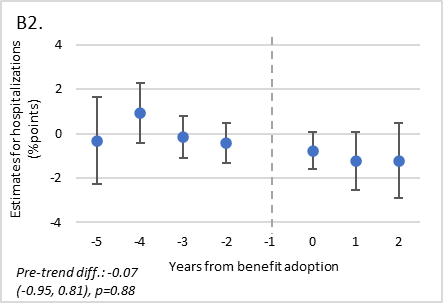

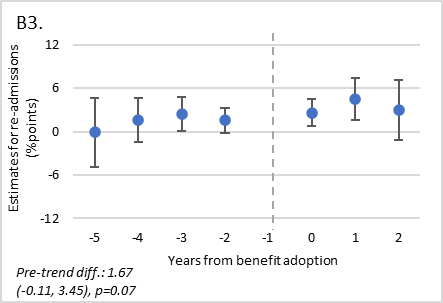


# Appendix Figure A8. Event-study plots from the analysis of any SSBCI among highly frail enrollees

| **Panel A. Non-dual eligible enrollees** |
| --- |
| 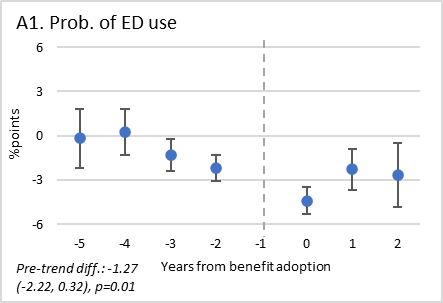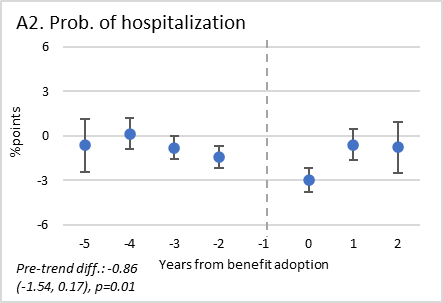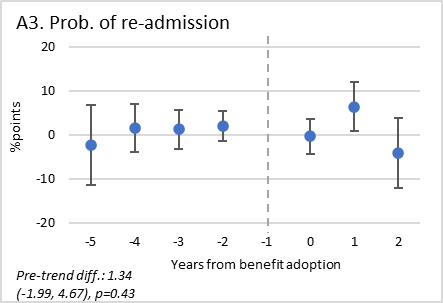 |
| **Panel B. Dual eligible enrollees** |
| 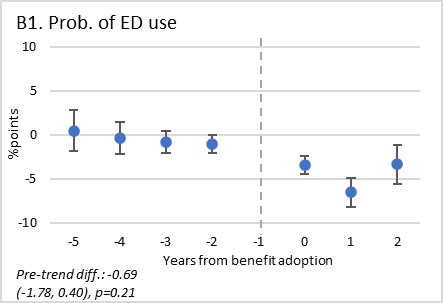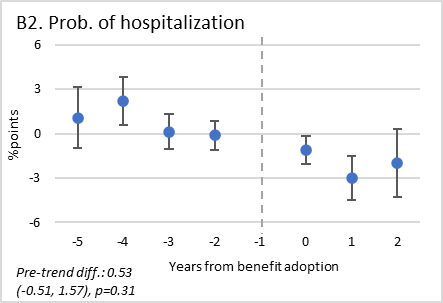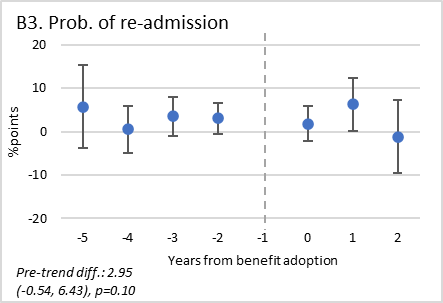 |

Abbreviations: SSBCI, special supplemental benefits for the chronically Ill; ED, emergency department; %points, percentage points; pre-trend diff., pre-treatment trends difference.

Notes: Pre-trend diff. reports the average differential change in the outcome between benefit-offering plans and non-offering plans during the pre-treatment period. The outcome was the annual probability of having an adverse health event (ED visit, hospitalization, or re-admission). The unit of Y-axis is one percentage point. 95% confidence intervals are in parentheses, and the p-value corresponds to the parallel trends test during the pre-treatment period. In graphs, error bars indicate the 95% CIs. Year −1 is the omitted reference period.

Appendix Figure A9. Event-study plots from the analysis of ≥ 2 SSBCI among highly frail enrollees

| **Panel A. Non-dual eligible enrollees** |
| --- |
| 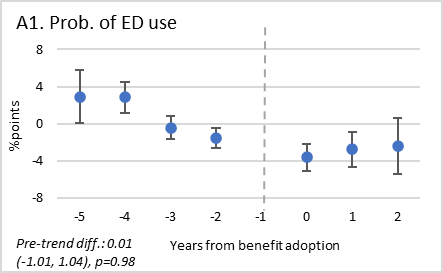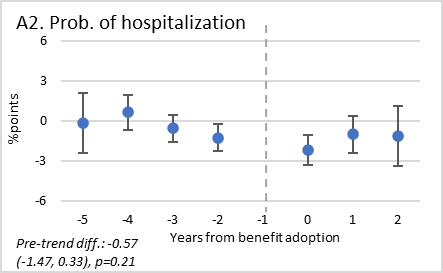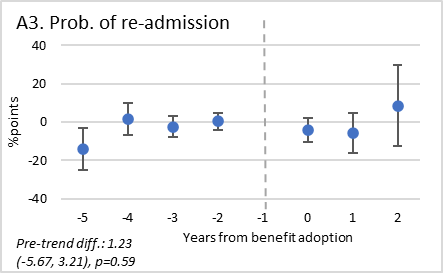 |
| **Panel B. Dual eligible enrollees** |
| 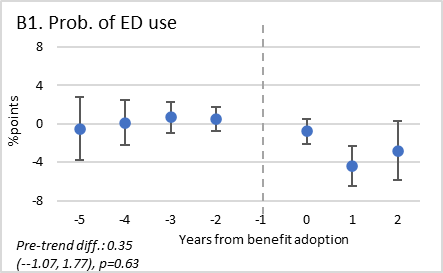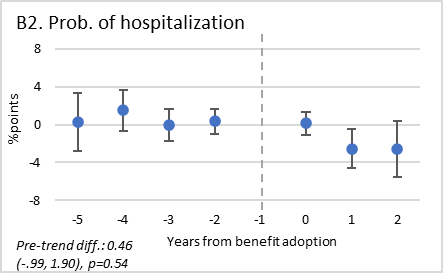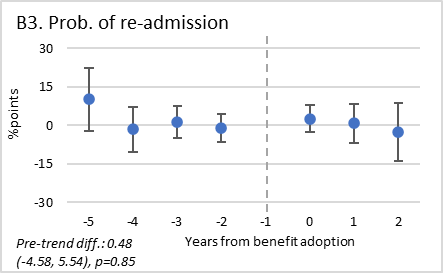 |

Abbreviations: SSBCI, special supplemental benefits for the chronically Ill; ED, emergency department; %points, percentage points; pre-trend diff., pre-treatment trends difference.

Notes: Pre-trend diff. reports the average differential change in the outcome between benefit-offering plans and non-offering plans during the pre-treatment period. The outcome was the annual probability of having an adverse health event (ED visit, hospitalization, or re-admission). The unit of Y-axis is one percentage point. 95% confidence intervals are in parentheses, and the p-value corresponds to the parallel trends test during the pre-treatment period. In graphs, error bars indicate the 95% CIs. Year −1 is the omitted reference period.

Appendix Figure A10. *HonestDiD* results for highly frail non-dual enrollees: Analysis of offering any expanded PHR benefit

A1. Prob. of ED use among highly frail non-dual enrollees A2. Prob. of hospitalization among highly frail non-dual enrollees


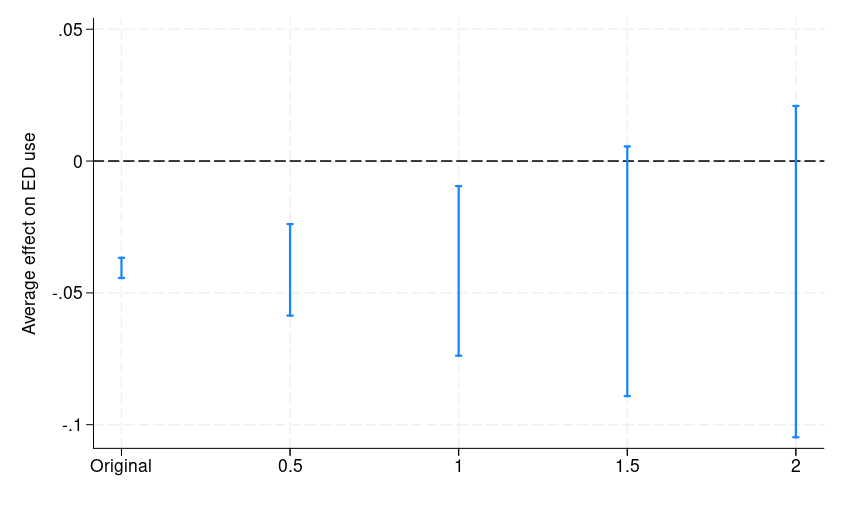

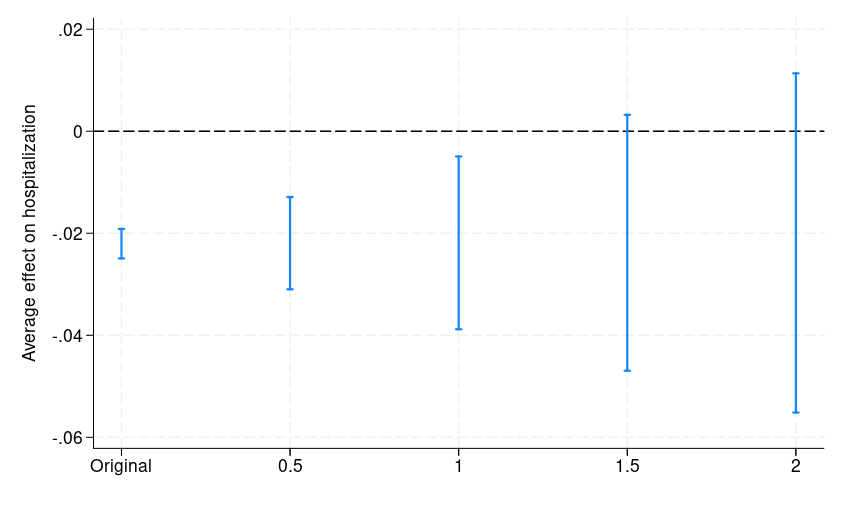


Abbreviations: PHR, primarily health-related; ED, emergency department.

Notes: Y-axis in the figures represents the annual probability of having an adverse health event (ED visit or hospitalization). Figures present the results from the HonestDiD method, which allows bounded deviations from parallel trends. Specifically, the method constrains post-treatment deviations to be bounded in magnitude relative to the deviations observed in the pre-treatment period and constructs confidence intervals that remain valid under these restrictions. The relative magnitude (M) to the pre-trend deviation is represented in the x-axis. For example, a value of 0.5 means that the post-treatment deviation from parallel trends is no greater than 50% of the pre-treatment period deviation; and a value of 1 means that the post-treatment deviation is no more than the pre-trend deviation. Each figure shows the average treatment effect under different assumptions about the relative magnitude bounds to deviations from parallel trends (M = 0.5, 1, 1.5, 2 times the maximum pre-trend deviation).

Appendix Figure A11. *HonestDiD* results for highly frail non-dual enrollees: Analysis of offering any SSBCI

A1. Prob. of ED use among highly frail non-dual enrollees A2. Prob. of hospitalization among highly frail non-dual enrollees


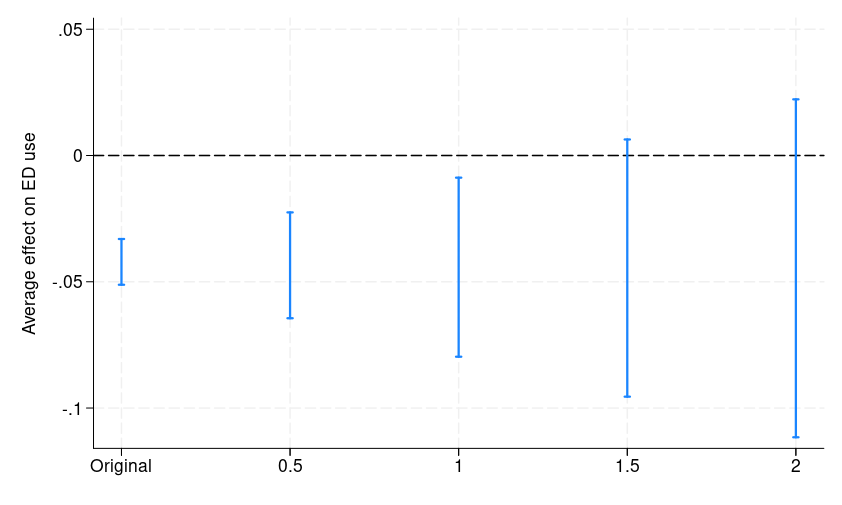

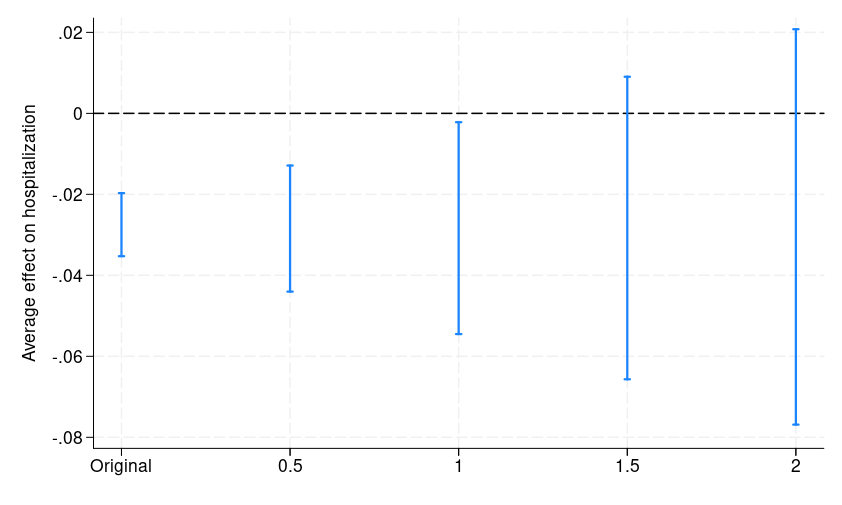


A1. ED use among highly frail dual-eligible enrollees A2. Hospitalization among highly frail dual-eligible enrollees

Abbreviations: SSBCI, special supplemental benefits for the chronically Ill; ED, emergency department.

Notes: Y-axis in the figures represents the annual probability of having an adverse health event (ED visit or hospitalization). Figures present the results from the HonestDiD method, which allows bounded deviations from parallel trends. Specifically, the method constrains post-treatment deviations to be bounded in magnitude relative to the deviations observed in the pre-treatment period and constructs confidence intervals that remain valid under these restrictions. The relative magnitude (M) to the pre-trend deviation is represented in the x-axis. For example, a value of 0.5 means that the post-treatment deviation from parallel trends is no greater than 50% of the pre-treatment period deviation; and a value of 1 means that the post-treatment deviation is no more than the pre-trend deviation. Each figure shows the average treatment effect under different assumptions about the relative magnitude bounds to deviations from parallel trends (M = 0.5, 1, 1.5, 2 times the maximum pre-trend deviation).

# Appendix Figure A12. *HonestDiD* results for highly frail dual-eligible enrollees: Analysis of offering any expanded PHR benefit

A1. Prob. of ED use among highly frail dual-eligible enrollees A2. Prob. of hospitalization among highly frail dual-eligible enrollees


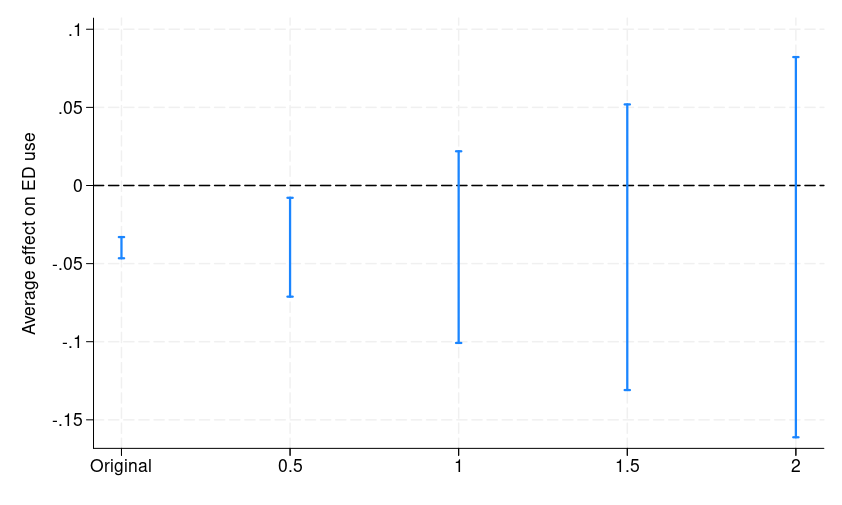

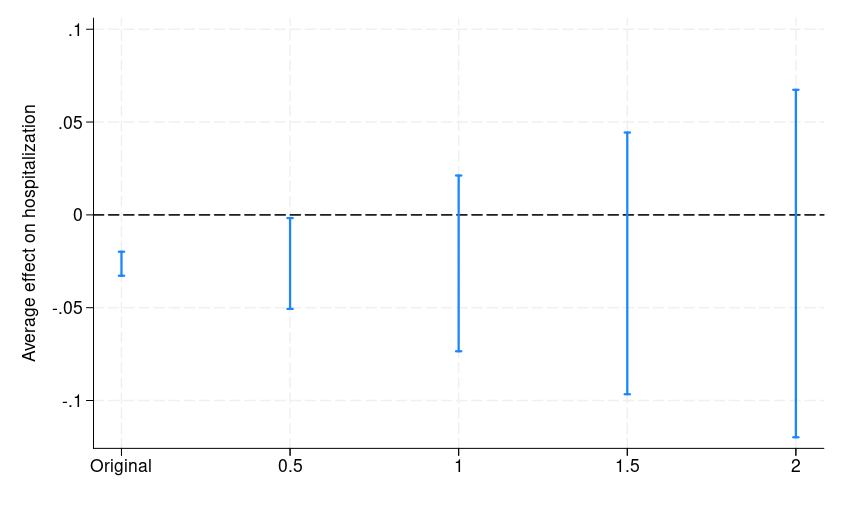


Abbreviations: PHR, primarily health-related; ED, emergency department.

Notes: Y-axis in the figures represents the annual probability of having an adverse health event (ED visit or hospitalization). Figures present the results from the HonestDiD method, which allows bounded deviations from parallel trends. Specifically, the method constrains post-treatment deviations to be bounded in magnitude relative to the deviations observed in the pre-treatment period and constructs confidence intervals that remain valid under these restrictions. The relative magnitude (M) to the pre-trend deviation is represented in the x-axis. For example, a value of 0.5 means that the post-treatment deviation from parallel trends is no greater than 50% of the pre-treatment period deviation; and a value of 1 means that the post-treatment deviation is no more than the pre-trend deviation. Each figure shows the average treatment effect under different assumptions about the relative magnitude bounds to deviations from parallel trends (M = 0.5, 1, 1.5, 2 times the maximum pre-trend deviation).
